# Incidence of lower extremity amputations in the diabetic compared with the non-diabetic population: A systematic review

**DOI:** 10.1371/journal.pone.0182081

**Published:** 2017-08-28

**Authors:** Maria Narres, Tatjana Kvitkina, Heiner Claessen, Sigrid Droste, Björn Schuster, Stephan Morbach, Gerhard Rümenapf, Kristien Van Acker, Andrea Icks

**Affiliations:** 1 Institute for Health Services Research and Health Economics, German Diabetes Center, Düsseldorf, Germany; 2 Institute for Health Services Research and Health Economics, Centre for Health and Society, Faculty of Medicine, Heinrich Heine University Düsseldorf, Germany; 3 German Center for Diabetes Research (DZD), Munich-Neuherberg, Germany; 4 Department of Diabetology and Angiology, Virgin Mary Hospital Soest, Germany; 5 Clinic for Vascular Surgery, Deaconess Foundation Hospital, Upper Rhine Vascular Center Speyer-Mannheim, Speyer, Germany; 6 Centre Santé des Fagnes, Chimay, Belgium; University of Colorado Boulder, UNITED STATES

## Abstract

Lower extremity amputation (LEA) in patients with diabetes results in high mortality, reduced quality of life, and increased medical costs. Exact data on incidences of LEA in diabetic and non-diabetic patients are important for improvements in preventative diabetic foot care, avoidance of fatal outcomes, as well as a solid basis for health policy and the economy. However, published data are conflicting, underlining the necessity for the present systematic review of population-based studies on incidence, relative risks and changes of amputation rates over time. It was conducted according to the Preferred Reporting Items for Systematic reviews and Meta-Analyses (PRISMA) guidelines. Nineteen out of 1582 studies retrieved were included in the analysis. The incidence of LEA in the diabetic population ranged from 78 to 704 per 100,000 person-years and the relative risks between diabetic and non-diabetic patients varied between 7.4 and 41.3. Study designs, statistical methods, definitions of major and minor amputations, as well as the methods to identify patients with diabetes differed greatly, explaining in part these considerable differences. Some studies found a decrease in incidence of LEA as well as relative risks over time. This obvious lack of evidence should be overcome by new studies using a standardized design with comparable methods and definitions.

**Systematic review registration number:**

PROSPERO CRD4201501780

## Introduction

The global prevalence of diabetes mellitus (DM) has risen to 8.8% in 2015, which corresponds to 415 million patients [1]. This leads to increasing numbers of individuals with diabetic foot disease, up to 75% of lower extremity amputations (LEAs) being performed in these patients [2, 3]. LEAs reduce the quality of life [4] and increase mortality [5–7] as well as medical costs [8]. Early initiatives [9–11] persued the goal to reduce the number of LEAs in diabetic patients. However, ensuing epidemiological studies showed marked variations in the incidence, relative risks and time trends of LEA compared with the non-diabetic population, owing to differences in study design and methodological approaches [3, 12–14]. Furthermore, there is no generally accepted definition of major or minor amputation [12, 15, 16] and the unambiguous identification of a diabetic person is conducted in a large variety of ways [16–19]. Also, incidence data differ largely depending on whether they are based on the number of amputations, hospitalizations or amputees. For example, the incidence rates per 10,000 diabetic patients per year were reported with 158 amputations, 101 hospitalizations and 87 amputees [20]. Finally, statistical methods differ largely between the studies, as they analysed crude or adjusted incidence rates. [13, 18, 21]. Reliable incidence rates of LEA in diabetic and non-diabetic populations are of utmost global importance for further improvements in the care of diabetic patients, the avoidance of fatal outcomes and for decisions relating to health policy and economy. Some reviews on this topic have been published [22–27]; however, they had some limitations with respect to definitions of LEA, at-risk population and statistical methods. Therefore, we felt that a systematic review was overdue. The aims of this systematic review were (1) to analyse the incidence of LEA in the population with and without diabetes as well as the corresponding relative risks, and (2) to investigate time trends.

## Methods

The systematic review was performed according to the Preferred Reporting Items for Systematic Reviews and Meta-Analyses (PRISMA) guidelines [28] (S1 Table). A study protocol with the registration number PROSPERO CRD42015017809 (S1 Text) has been published [29].

### Search strategies and selection criteria

Literature was searched systematically in the international biomedical literature databases MEDLINE, EMBASE, Web of Knowledge and publisher databases Journals@OVID and ScienceDirect until December 2014 with no retroactive time limit. Database-specific controlled terms (MeSH, EMTREE) and additional free-text terms were used. The search terms (combined by Boolean operators) were amputation, amputee (search component “intervention”), lower extremity, foot, feet, limb, etc. (search component “problem”) and epidemiology, prevalence, incidence, frequency, population survey, survey data, administrative data, community data etc. (search component “epidemiologic studies”). The systematic search is based on a linear block-building model. Some cherry-picking strategies were added. Potentially eligible studies in reference lists of review articles and relevant studies were identified by additional handsearch. The detailed search strategies are provided as supporting information (S2 Text).

Full-text articles were included if they met inclusion criteria regarding types of studies, types of population and the main outcome regardless of the type of amputations, age, sex and ethnicity.

#### Types of studies

All population-based studies analysing incidence rates in the diabetic versus the non-diabetic population with reported relative risks were included.

#### Types of population

(1) The population at risk had to be defined by official statistics, which means nationwide data or for example all inhabitants of a defined region or all insurants of a statutory health insurance. (2) The diabetic population had to be precisely described (e.g. register, estimation based on age–sex-specific prevalence data). Individuals without diabetes were considered with the aim of comparing incidences between the diabetic and non-diabetic populations.

#### Outcomes (definition of LEA, epidemiological measures)

The main outcome was analysed depending on the level of amputation: I. any LEA (both major and minor amputations); II. major amputations; III. minor amputations. This was always done in terms of reporting the incidence of LEA: a) person level: only one amputation per person (first or highest); b) case level: data based on hospital discharge rates. This may be several hospitalizations per person in the same calendar year; nevertheless, as a rule only one amputation per person per admission is considered; c) procedure level: all amputations per person are taken into account.

The epidemiological measure of the main outcome was the incidence rate (IR) of LEA among patients with diabetes and among persons without diabetes. In order to compare incidence rates of LEA between the diabetic and non-diabetic population, the relative risks (RRs) were taken into account. Furthermore the attributable risk (AR) and the population attributable risk (PAR) were considered where available. AR is the proportion of LEA among persons with diabetes that is attributable to diabetes. PAR is the proportion of LEA in the whole population that is attributable to diabetes.

Studies were excluded if: (a) they solely reported incidences of LEA among persons with diabetes without comparison to non-diabetic persons; (b) incidence rates were reported in relation to the total (diabetic and non-diabetic) population and not exclusively using the diabetic population as a population at risk; (c) exclusively crude incidence rates were reported; (d) studies were published in a language other than English.

### Data collection and extraction

Four authors (H.C., A.I., T.K. and B.S.) independently screened all the retrieved titles and abstracts to identify potentially eligible articles. Full-text screening was performed by four authors (M.N., H.C., T.K. and B.S.). Disagreements were resolved by discussion. Data extraction was performed by M.N., H.C. and B.S., including information about first author, publication year, country, study period, study design, study populations (diabetic and non-diabetic), definition of LEA and study results. The reported IR was recalculated as IR per 100,000 PY if not originally reported as such.

### Quality assessment

The quality of the eligible studies was assessed by two independent reviewers (M.N. and B.S.) in consideration of study limitations, risk of bias and the degree of imprecision (missing information according to absolute number of cases or missing confidence interval) using the modified checklist (S2 Table) adapted to Methodological Evaluation of Observational Research (MORE) [30], the Scottish Intercollegiate Guidelines Network (SIGN) [31], and the Cochrane approach Study Quality Guide [32]. This tool was used to define criteria based on clinical and epidemiological expertise and studies with quality ranked as high, acceptable or low according to the recommendations of SIGN [31]. Detailed information can be found in the study protocol [29]. Only studies with high or acceptable quality were included in the review.

### Statistical methods

The results (IR, RR, AR, and PAR) from included studies were presented as age–sex-adjusted estimates. All estimates were presented with 95% confidence intervals (95% CI) if available. In either case, the number of events of LEA was included in the results tables. We described time trend as “descriptive” if the reviewed studies only reported annual incidence of LEA without using appropriate multivariate regression models. Due to the high heterogeneity of the included studies no meta-analysis was performed.

## Results

**Fig 1:**
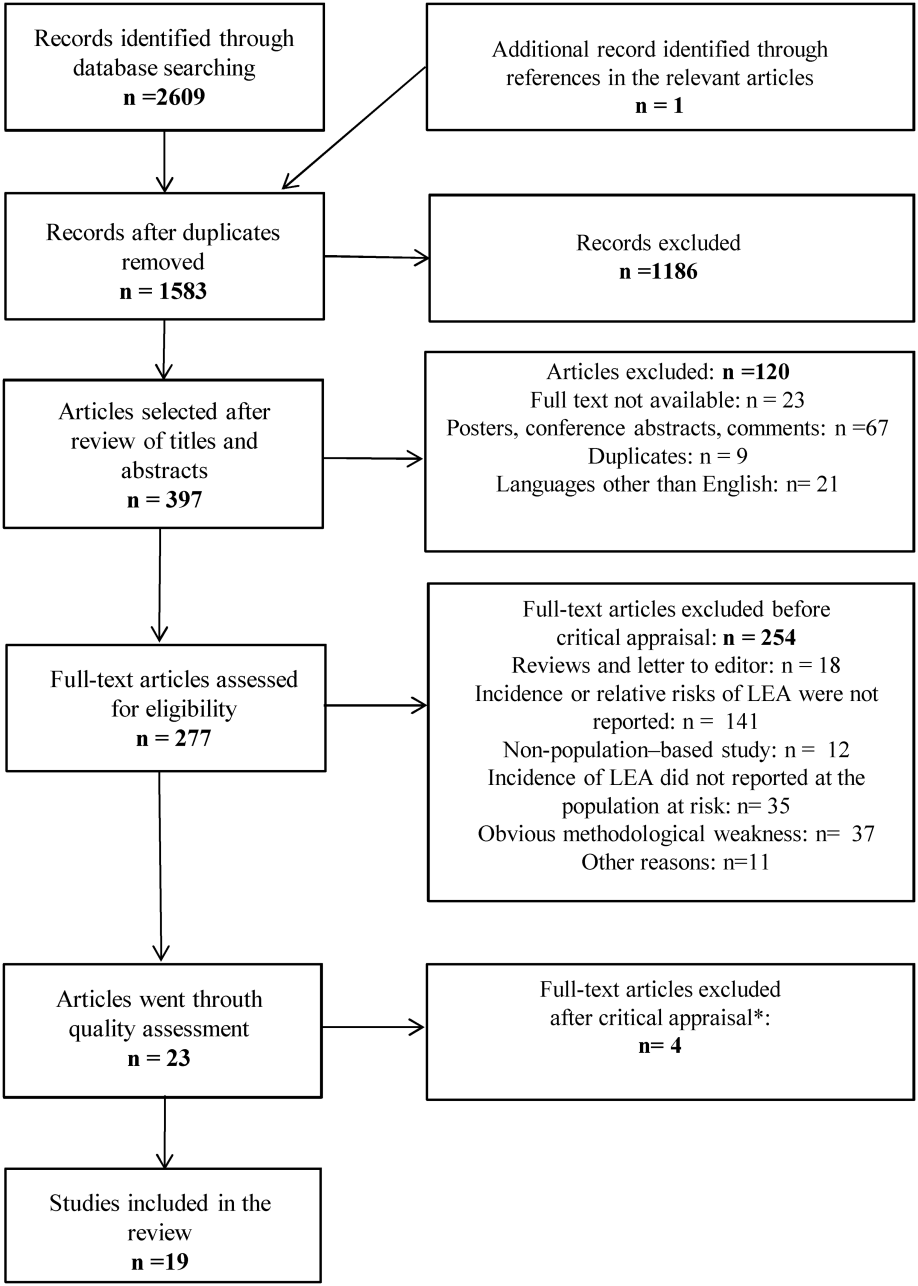
Flowchart of the systematic review process. * The quality of the eligible studies was assessed using the modified checklist (s. S2 Table).

1582 citations were initially retrieved, from which 19 papers were included in this review. The selection procedure is presented in Fig 1.

### Study design

The included studies used different sources to estimate the diabetic population at risk: eight studies used data from national surveys [11, 13, 18, 19, 33–36], seven used data from national or local diabetes registries or linked data from several diabetes-related data sources [2, 12, 16, 17, 37–39], and four adopted diabetes prevalence data from other studies [3, 15, 40, 41]. Regarding the diabetes population at risk, most studies used patients with diagnosed diabetes as a denominator, two studied persons with treated diabetes [17, 18], and one studied [15] individuals with diagnosed and non-diagnosed diabetes (Table 1).

**Table 1:** Incidence of LEA in the diabetic compared with the non-diabetic populations—Study characteristics.

Study	Study period, design and population	Diabetic population at risk, number at risk (n)	Data sources for diabetic prevalence	Definition of LEA by anatomical level	Counting of LEA	Cause for LEA	Data sources for LEA	Time trend
Gujral et al., 1993, UK[40]	1980–1985 Population of Leicestershire n = 850,000	Known DM	According to the study by Samanta A.[42]	Any	One per person (first)	Non-traumatic non-tumour	Discharge data supplied by the Hospital Activity Analysis	NA
Siitonen et al., 1993, Finland[37]	1978–1984 Inhabitants of Kuopio province in eastern Finland 1978 n = 253,157	Known DM n = 7,636	Register for drug-treated patients and survey for diet-treated patients	Any	One per person (first)	Only LEA attributable to PVD: non-traumatic, non-tumour, non-frostbite	The registers of the operating theatres of all five hospitals with facilities for amputation in the study area	NA
Van Houtum & Lavery, 1996, Netherlands and USA[34]	1991 Netherlands: nationwide USA: State of California with the exception of Veteran Administration hospitals and military facilities	Known DM	Netherlands: Central Bureau for Statistics California: National/Hispanic Health and Nutrition Examination Survey	Any	Hospital discharge rate	Non-traumatic	Netherlands: SIG Health Care Information California: Office of Statewide Planning and Development	NA
Van Houtum et al., 1996, Netherlands[36]	1992 Netherlands: nationwide	Known DM	Central Bureau for Statistics	Any	Hospital discharge rate	Non-traumatic	SIG Health Care Information	NA
Van Houtum & Lavery, 1996, Netherlands[35]	1991–1992 Netherlands: nationwide	Known DM	Central Bureau for Statistics	Any	Hospital discharge rate	Non-traumatic	SIG Health Care Information	NA
Lavery et al., 1996, USA[11]	1991 State of California with the exception of Veteran Administration hospitals and military facilities	Known DM	National/Hispanic Health and Nutrition Examination Survey	Any	Hospital discharge rate	Non-traumatic	Office of Statewide Planning and Development	NA
Morris et al., 1998, UK[39]	1993–1994 Residents of Tayside who were registered with a Tayside general practitioner n = 364,880	Known DM n = 7,079	Diabetic patients were identified by the DARTS / Medicines Monitoring Unit (MEMO) Collaboration by linkage of eight diabetes-related data sources	Any Major: any LEA through or proximal to the ankle joint Minor	One per person (first)	Non-traumatic non-tumour	The primary data source was the Scottish Morbidity Record 1 (SMR1) database based on patient discharges	NA
Calle-Pascual et al., 2001, Spain[15]	1989–1999 Residents of area 7 in Madrid 1991 n = 569,307	Known & unknown DM n = 37,932	According to the Lejona study[43]	Major: through or proximal to the tarsometatarsal joint (GLEAS protocol) Minor	One per person (first); all	Non-traumatic	Operating theatre records; secondary sources used were vascular service department and endocrinology service discharge records	+
Trautner et al., 2001, Germany[2]	1990–1991, 1994–1998 Residents of the city of Leverkusen 1990 n = 160,684	Known DM	East German diabetes registry	Any Major: any LEA above the ankle	One per person (first)	Non-traumatic	Operating theatre documentation	+
Wrobel JS et al., 2001, USA[41]	1996–1997 Medicare population aged ≥ 65 years 1996 n = 56,453,929	Known DM 1996 n = 6,037,804	The regional prevalence of DM was based on HEDIS (Healthcare Effectiveness Data and Information Set) 3.0 using Medicare Part B claims data for 1995–1996	Major: transtibial or transfemoral LEA	Hospital discharge rate	Non-traumatic	Hospital discharges from the MEDPAR file for 1996–1997	NA
Trautner et al., 2007, Germany[16]	1990–1991, 1994–2005 Residents of the city of Leverkusen 1990 n = 160,684	Known DM	East German diabetes registry	Any Major: any LEA above the ankle	One per person (first)	Non-traumatic	Operating theatre documentation	+
Canavan et al., 2008, UK[38]	1995–2000 Residents of the South Tees area (area of high long-term unemployment) 2001 n = 273,987	Known DM	Community diabetes registry	Any Major: through or proximal to the tarsometatarsal joint (GLEAS protocol) Minor	Any: all LEAs Major: One per person (first); all	Non-traumatic	Operating theatre records, limb-fitting centre records, and hospital discharge data	+
Fosse et al., 2009, France[18]	2003 Nationwide	Treated DM	According to the study by Kusnik-Joinville et al.[44]	Any	One per person (highest)	All LEA; non-traumatic	French national hospital discharge database	NA
Icks et al., 2009, Germany[12]	2005–2007 All insured persons of one statutory health insurance n = 1,580,744	Known DM n = 87,288	East German diabetes registry	Any Major: any LEA proximal to the midtarsal level, according to the 2007 International Consensus of the Diabetic Foot, Boulton, 2008)	One per person (first)	Non-traumatic	One statutory health insurance company, using OPS codes from hospital discharge documentation	NA
Ikonen et al., 2010, Finland[17]	1997–2007 Nationwide, total population > 5 million	Predominant treated DM n = 396,317 of which 50,027 persons with ITDM and 346,290 with NITDM	FinDM II database, which includes: register of individuals eligible for elevated reimbursement of medication for chronic conditions including DM; prescription register; national hospital discharge register; cause-of-death register; medical birth register	Major: any LEA through or proximal to the ankle joint	One per person (first)	All	Hospital discharge register	+
Almaraz et al., 2012, Spain[3]	1998–2006 Residents of Andalusia aged ≥ 30 years 2006 n = 7,975,672	Known DM 2006 n = 281,632	According to the DECODE Study[45]	Any Major: through or proximal to the ankle joint Minor	Hospital discharge rate	Non-traumatic non-tumour	The CMBD information system (Conjunto mínimo básico de datos, a basic set of data)	+
Buckley et al., 2012, Ireland[33]	2005–2009 Nationwide population aged ≥ 20 years 2005 n = 2,987,595 2009 n = 3,242,920	Known DM 2005 n = 137,554 2009 n = 151,698	The prevalence of diabetes in the population in 2007 from the Institute of Public Health, Ireland	Any Major: through or proximal to the ankle joint Minor	Hospital discharge rate	Non-traumatic	Data on hospital discharges from the Hospital In-Patient Enquiry (HIPE)	+
Gregg et al., 2014, USA[19]	1990–2010, Nationwide, overall U.S. population aged ≥ 20 years	Known DM 1990 n = 6,536,163 2010 n = 20,676,427	National Health Interview Survey (NHIS)	Any	Hospital discharge rate	Non-traumatic	National Hospital Discharge Survey (NHDS)	+
Lombardo et al., 2014, Italy[13]	2001–2010 Nationwide	Known DM 2012 n = ca. 3 Mio	Italian National Institute of Statistics	Any Major: any LEA above the ankle joint Minor	One per person (highest); hospital discharge rate	Non-traumatic non-tumour	National Hospital Discharge Record Database	+

DPAR diabetic population at risk; LEA lower extremity amputation; AR attributable risk; PAR population attributable risk; DD diabetes duration;

DM diabetes mellitus; ITDM insulin-treated diabetes mellitus; NITDM non-insulin–treated diabetes mellitus; PVD peripheral vascular disease;

GLEAS Global Lower Extremity Amputation Study [46].

With regard to the main outcome, different definitions of LEA were considered: eight studies estimated incidence of LEA counting one amputation per person [2, 12, 16–18, 37, 39, 40], of which, five presented data stratified by amputation level [2, 12, 16, 17, 39]; eight analysed incidence based on hospital discharge data [3, 11, 19, 33–36, 41], including three studies that presented data stratified by amputation level [3, 33, 41]; three studies combined different methods for counting amputations [13, 15, 38]. The majority of the studies estimated non-traumatic LEAs, five described non-traumatic and non-tumour–related LEAs [3, 13, 37, 39, 40], and two reported incidence of LEAs independent of their cause [17, 18]. Regarding patient-related parameters, the majority of studies reported patient age at the time of amputation. In contrast, the duration of diabetes at the time of amputation was reported in only four studies [2, 16, 37, 39]. Likewise, comorbidity and in particular mortality, was described in only a few studies [18, 33, 37]. With regard to statistical significance, more than half (n = 10) of all studies reported incidence of LEAs with a 95% confidence interval. Nine studies analysed time trends [2, 3, 13, 15–17, 19, 33, 38], seven of which with appropriate statistical methods and two only descriptively [15, 38] (Table 1).

### Quality assessment

Using the “Methodology Checklist critical appraisal” tool we considered 10 studies to be of high quality, nine to be of acceptable quality, and four studies to be of low quality. The latter were excluded (Fig 1). The important critical points were: the unclear definition of LEA, the missing data concerning confidence intervals, and the absolute number of LEAs.

### Main findings

#### I.a. Incidence of any LEA: One LEA per person

A total of eight studies estimated any LEA counting one amputation per person [2, 12, 13, 16, 18, 37, 39, 40]. The proportion of LEAs conducted among diabetic persons varied strongly from 27% [39] to 75.5% [2] (Table 2).

**Table 2:** Incidence of LEA in the diabetic compared with the non-diabetic populations—Results.

Study	Number of LEA, age, DD in diabetic/non-diabetic population	IR* (95% CI) in diabetic population—total	IR* (95% CI) in non-diabetic population—total	IR* (95% CI) in diabetic population—stratified by sex and ethnic origin	IR* (95% CI) in non-diabetic population—stratified by sex and ethnic origin	RRs (95% CI)—total population	RRs (95% CI)—stratified by sex and ethnic origin	AR (%, (95% CI)) PAR (%, (95% CI))	Time trend
**I. Incidence of any LEA**									
*I*.*a*. *Incidence of any LEA*: *One LEA per person*									
Gujral et al., 1993, UK[40]	DM: 269 (including 3 LEA among Asian) non-DM: NA	NA	NA	White: 142 (126–159) Asian: 34 (11–107) White men: 175 (151–204) White women: 108 (89–128) Asian men: 68 (55–214) Asian women: 0	White: 15 (14–16) Asian: 4 (2–6) White men: 17 (15–19) White women: 13 (12–15) Asian men: 5 (13–10)# Asian women: 2 (1–4)	NA	White: 9.5† White men: 10.3† White women: 8.3† Estimation using quotient method§	NA	NA
Siitonen et al., 1993, Finland[37]	During the entire study period total LEA: 477 DM: 254 (53.2%) mean age men: 67.1 Y (SD 10.0) women:73.8 Y (SD 9.1) DD men: 13 Y (SD 8.2); women:12.0 Y (SD 8.1) non-DM: 223 mean age men: 75.5 Y (SD 20.9) women:78.3 Y (SD 10.7)	NA	NA	Men: 349.1 Women: 239.4	Men: 33.9 Women: 17.2	NA	Men: 10.3 Women: 13.8 Estimation using quotient method§	NA	NA
Morris et al., 1998, UK[39]	During the entire study period total LEA: 192 DM: 52 (27%) median age:70 (34–88) Y men: 68 (34–88) Y women: 74 (52–87) Y median DD by cases 6 Y non-DM: 140 median age: 71 (14–95) Y men 68 (14–93)Y women: 74 (15–95) Y	247.91	20.1	Men: 280.8 Women: 188.02	Men: 23.07 Women: 17.36	12.33 (8.64–17.52) Estimation using quotient method§	Men: 12.17 (8.74–16.95) Women: 10.83 (7.32–16.03) Estimation using quotient method§	AR total: 92 Men: 92.11 Women: 91.49 PAR total:25.64 Men: 30.74 Women: 17.99	NA
Trautner et al., 2001, Germany[2]	During the entire study period total LEA: 339 mean age: men: 68.6 Y (SD 10.6) women: 74.5 Y (SD 10.1) DM: 256 (75.5%) Mean DD: 16.1 Y (SD 10.3), median 15 Y range (0–55) non-DM: 83	1990: 224 (136–311) 1991: 143 (75–210) 1994: 226 (141–312) 1995: 175 (96–255) 1996: 180 (101–259) 1997: 455 (0–989) 1998: 195 (113–278) Total study duration: 230 (150–311)	1990: 7 (2–12) 1991: 10 (5–16) 1994: 12 (6–18) 1995: 11 (5–16) 1996: 2 (0–5) 1997: 10 (5–15) 1998: 8 (4–13) Total study duration: 8 (4–13)	Men: 311 (150–472) Women: 154 (117–192)	NA	26 (17–39) Estimation using quotient method§ 20.5 (15.84–26.81) Estimation using Poisson model	NA	AR 96 (94–97) PAR 70 (61–77)	**IR**: Diab and non-diab pop combined: unchanged: RR per calendar year 0.99 (0.95–1.03), p = 0.51 **RRs**: NA
Trautner et al., 2007, Germany[16]	During the entire study period total LEA: 692 mean age: men: 69.0 Y (SD 10.4) women: 75.4 Y (SD 10.7) DM: 501 (72.4%) Mean DD: 15.1 Y (SD 10.7), median 14 range (0–61) non-DM: 191	1990–1998 s. Trautner 2001 1999: 191 (113–269) 2000: 165 (93–237) 2001: 78 (48–107) 2002: 131 (67–195) 2003: 119 (67–171) 2004: 113 (52–174) 2005: 235 (136–335)	1990–1998 s. Trautner 2001 1999:7 (3–10) 2000:8 (3–13) 2001: 8 (4–13) 2002:4 (1–7) 2003: 13 (7–18) 2004: 12 (7–17) 2005: 12 (7–17)	NA	NA	25.685 (17.731–37.787) Estimation using Poisson model	NA	AR varied between 99 in 1996 and 89 in 2003 and 2004 The PAR varied between 90 in 1996 and 49 in 2004	**IR**: DM pop.: reduced: RR per calendar year 0.976 (0.958–0.996), p = 0.0164; non-DM pop: unchanged: RR per calendar year 1.022 (0.989–1.056), p = 0.158 **RRs**: decreased: RR per calendar year 0.95 (0.914–0.986), p = 0.0078
Fosse et al., 2009, France[18]	During the entire study period total LEA: 15,353 DM: 7,955 (52%) mean age 70 Y (SD 11) non-DM: 7,398 mean age 69 Y (SD 20)	158.0 (traumatic LEA included) 147.0 (non-traumatic LEA)	13.4 (traumatic LEA included) 11.4 (non-traumatic LEA)	NA	NA	11.8 ((11.0–12.6) traumatic LEA included) 12.9 ((12.0–13.9) non-traumatic LEA) Estimation using quotient method§	NA	NA	NA
Icks et al., 2009, Germany[12]	Between 2005–2007 total LEA: 994 DM: 652 (66%) mean age men: 66.6 Y (SD 10.5) women: 70.0 Y (SD 12.4) non-DM: 342 mean age: men: 64.2 Y (SD 14.9) women: 67.8 Y (SD 18.2)	121.2 (108.6–133.7)	16.4 (14.3–18.5)	Men: 176.5 (156.0–196.9) Women: 76.9 (61.9–91.8)	Men: 20.0 (17.0–23.1) Women: 13.4 (10.7–16.2)	7.4 (6.3–8.7) Estimation using quotient method§	Men: 8.8 (7.3–10.7) Women: 5.7 (4.3–7.6) Estimation using quotient method§	AR total: 86 (84–89) Men: 89 (86–91) Women: 83 (77–87) PAR total: 51 (46–56) Men: 59 (54–64) Women: 40 (30–48)	NA
Lombardo et al, 2014, Italy[13]	Between 2001–2010, a mean annual number of total LEA: 11,639 DM: 6,823 (58,6%) mean age: 71.1 Y (SD 11.1) non-DM: 4,816 mean age: 73.2 Y (SD 17.9)	2003: 149.8 2004: 150.8 2005: 142.3 2006: 139.8 2007: 134.3 2008: 125.4 2009: 128.3 2010: 128.7	2003 9.2 2004 9.0 2005 8.9 2006 9.1 2007 8.8 2008 9.1 2009 8.8 2010 8.6	NA	NA	RR for the whole period 10.95 (9.37–12.81) Estimation using Poisson model	NA	NA	**IR**: Entire pop. (both diab. and non-diab pop.) unchanged: RR per year 0.98 (0.96–1.01), p = 0.203 **RRs**: unchanged
*I*.*b*. *Incidence of any LEA*: *Hospital discharge rates*									
Van Houtum & Lavery, 1996, Netherlands and USA [34]	Netherlands: DM: 1,558 hospitalizations (47.8%) average age: 71.1 Y (SD 11.8) California: DM:5,283 (62.9%) hospitalizations average age 64.8 Y (SD 13.2)	Netherlands: 361 California: 499	NA	Netherlands: Men: 469 Women: 298 (RR men vs women 1.6) California: Men: 753 Women: 321 (RR men vs women 2.4)	NA	Netherlands: 19.7 California: 23.7 Estimation using quotient method§	NA	NA	NA
Van Houtum et al., 1996, Netherlands [36]	Total 3,335 hospitalizations: DM: 1,575 (47%) mean age: 70.9 Y (SD 11.8) non-DM: 1760† mean age 68.4 Y (SD 19.8)	251.7 (99.9% CI 214.6–288.8)	12.4 (99% CI 11.4–13.3)	Men: 355.1 (99.9% CI 294.1–416.1) Women: 174.3 (99.9% CI 133.1–215.6) (RR men vs women 2.04; 99.9% CI 1.69–2.45)	Men: 17.3 (99.9% CI 15.5–19.1) Women: 8.8 (99.9% CI 7.7–9.9)	20.3 (99.9% CI 18.5–22.5) Estimation using quotient method§	Men: 20.5 (99.9% CI 18.1–23.3) Women: 19.8 (99.9% CI 17.2–22.9) Estimation using quotient method§	NA	NA
Van Houtum & Lavery, 1996, Netherlands[35]	Between 1991–1992 total 6,665 hospitalizations: DM: 3,127 (47%)	Nationwide: 250.5 Regional: 101.5–446.4	Nationwide: 12.4 Regional: 7.7–17.7	Nationwide: Men: 336.8 Women: 191.9 (RR men vs women 1.8) Regional: Men: 128.2–534.2 Women: 57.2–591.8 (RR men vs women: 1.14–4.59)	Nationwide: Men: 17 Women: 9 Regional: Men: 12.6–24.9 Women: 4.1–15.6	Nationwide: 20.2 Regional: 7.87–45.17 Estimation using quotient method§	NA	NA	NA
Lavery et al., 1996, USA[11]	Total: 8,169 hospitalizations DM: 5,114† (62.6%)	539.0 (519.0–559.0)	NA	Non-Hispanic white: 559.8 (528.5–591.0) Hispanic: 444.3 (416.4–472.1) African-American: 952.5 (877.9–1027.1)	Non-Hispanic white: 20.1 (19.2–20.9) Hispanic: 17.4 (15.1–19.6) African-American: 67.9 (60.8–72.9)	NA	Non-Hispanic white: 27.84 (25.35–30.33) Hispanic: 25.56 (22.13–29.51) African-American: 14.16 (12.57–15.95) Estimation using quotient method§	NA	NA
Almaraz et al., 2012, Spain[3]	Between 1998–2006 total hospitalizations 16, 210: DM: 11,770 (72.6%) mean age: 70.3 Y (SD 10.7) men: 68.5 Y (SD 10.7) women: 74.4 Y (SD 11.0) non-DM: 4,440 mean age: 71.3 Y (SD 13.7) men: 69.0 Y (SD 13.2) women: 77.6 Y (SD 13.2)	1998–2000: 301.7 (274–328.9) 2001–2003: 322.8 (295.0–350.7) 2004–2006: 344.0 (315.4–372.4)	1998–2000: 9.6 (8.7–10.5) 2001–2003: 9.7 (8.9–10.6) 2004–2006: 8.3 (7.6–9.1)	Men:1998–2000: 466.6 (417.0–516.4) 2001–2003: 514.1 (463.2–564.9) 2004–2006: 582.8 (529.8–635.6) Women: 1998–2000: 171.3 (146.5–196.1) 2001–2003: 166.9 (141.1–192.6) 2004–2006: 144.9 (120.9–186.9)	Men: 1998–2000: 16.3 (14.5–18.0) 2001–2003: 16.5 (14.9–17.9) 2004–2006: 13.6 (12.1–15.1) Women: 1998–2000: 3.9 (3.2–4.7) 2001–2003: 4.1 (3.3–4.8) 2004–2006: 3.8 (3.1–4.5)	1998–2000: 31.4 (27.7–34.2) 2001–2003: 33.1 (29.7–38.0) 2004–2006: 41.3 (37.0–45.1) Estimation using quotient method§	Men: 1998–2000: 28.7 (25.2–32.7) 2001–2003: 31.0 (27.2–38.4) 2004–2006: 42.9 (37.6–48.3) Women: 1998–2000: 43.2 (34.9–52.6) 2001–2003: 41.6 (33.9–50.3) 2004–2006: 38.1 (31.0–34.5) Estimation using quotient method§	NA	**IR**: DM pop.: increased: RR per calendar year 1.009 (1.002–1.016), p = 0.016 non-DM pop. reduced: RR per calendar year 0.974 (0.964–0.986), p<0.01 **RRs**: increased by 31.6%; men: increased by 49.4%; women: decreased by 11.9%
Buckley et al., 2012, Ireland[33]	During the entire study period total hospitalizations: 2,776 DM: 1,654 (53.5%^#^) non-DM: NA	2005: 144.2 (123.2–166.9) 2009:175.7 (152.3–200.9)	2005: 12.0 (10.7–13.5) 2009: 9.2 (8.1–10.4)	NA	NA	2005: 22.3 (19.1–26.1) 2006: 21 (17.8–24.7) 2007: 21.9 (18.8–25.6) 2008: 22 (18.9–25.6) 2009: 29.2 (24.9–34.3) Estimation using Poisson model	NA	NA	**IR**: DM pop.: increased non-significantly#, p = 0.11 non-DM pop.: decreased non-significantly#, p = 0.16 **RRs**: unchanged p = 0.4
Gregg et al., 2014, USA[19]	DM Pop: n 1990 = 50,364 n 1995 = 76,531 n 2000 = 80,658 n 2005 = 69,074 n 2010 = 73,067	1990: 584 (493–674) 1995: 704 (591–817) 2000: 487 (416–559) 2005: 355 (309–401) 2010: 284 (194–373)	1990: 31 (27–35) 2000: 27 (23–31) 2010: 27 (19–35)	Men: 1990: 807 (627–988) 2000: 620 (503–737) 2010: 388 (251–525) Women:1990: 416 (333–499) 2000: 268 (256–280) 2010: 182 (171–192) White: 1990: 418 (331–505) 2000: 362 (297–427) 2010: 172 (102–241) Black: 1990: 981 (654–1307) 2000: 522 (407–638) 2010: 400 (181–619)	NA	1990: 18.8 (15.1–22.6) 2000: 18.0 (14.3–21.7) 2010: 10.5 (6.0–15.0) Estimation using quotient method§	NA	NA	**IR**: DM pop.: reduced, p<0.001; non DM pop.: decreased non-significantly# **RRs**: decreased (descriptive)
Lombardo et al., 2014, Italy[13]	Between 2001–2010, a mean annual number of hospitalizations 13,581 DM: 8,232† (60.7%) non-DM: 5,339†	2003: 178.0 2004: 179.3 2005: 170.5 2006: 166.1 2007: 159.3 2008: 150.0 2009: 152.3 2010: 153.0	2003:10.1 2004: 9.8 2005: 9.8 2006: 9.9 2007: 9.5 2008: 9.9 2009: 9.6 2010: 9.4	NA	NA	NA	NA	NA	**IR**: descriptive **RRs**: NA
*I*.*c*. *Incidence of any LEA*: *All LEAs per person*									
Canavan et al., 2008, UK[38]	Between 1995–2000 total 454 LEA DM: 223 (49.1%)	1995–1996: 564.3 1999–2000: 176.0	1995–1996: 12.3 1999–2000: 22.8	NA	NA	1995–1996: 46 (25.7–90.6) 1999–2000: 7.7 (4.99–12.9) Estimation using quotient method§	NA	NA	**IR**: DM pop.: descriptive reduction#; non-DM pop.: descriptive increase# **RRs**: descriptive reduction
**II. Incidence of major LEA**									
*II*.*a*. *Incidence of major LEA*: *One LEA per person*									
Morris et al., 1998, UK[39]	During the entire study period total: 105† LEA DM 29† (27.6%†) non-DM: 76†	184.66	14.48	Men: 200.59 Women: 128.06	Men: 13.33 Women: 16.58	12.75 (8.43–19.29) Estimation using quotient method§	Men: 15.05 (9.94–22.79) Women: 7.76 (5.04–11.95) Estimation using quotient method§	NA	NA
Calle-Pascual et al., 2001, Spain[15]	NA	NA	NA	Men: 1989–1993: 67.1 (60.9–73.3) 1994–1996: 36.9 1997–1999 12.3 (10.5–14.1) Women:1989–1993: 13.3 (11.6–15.0) 1994–1996: 7.9 1997–1999 5.6 (4.9–6.3)	Men: 1989–1993 2.6 (2.2–3.0) 1994–1996: 1.1 1997–1999: 1.1 (0.4–1.8) Women: 1989–1993: 1.3 (1.1–1.5) 1994–1996: 0.4 1997–1999: 0.5 (0.4–0.6)	NA	1989–1993 Men: 25.8† Women: 10.2† 1994–1996: Men: 33.5† Women: 19.8† 1997–1999: Men: 11.2† Women: 11.2† Estimation using quotient method§	NA	**IR**: DM pop.: descriptive reduction#; non-DM pop.: descriptive reduction# **RRs**: NA
Trautner et al., 2001, Germany[2]	During the entire study period total: 157† LEA	79 (62–97)	NA	NA	NA	NA	NA	NA	**IR**: DM pop.: unchanged: RR per calendar year 0.991 (0.93–1.058), p = 0.78 non-DM pop.: unchanged **RRs**: NA
Trautner et al., 2007, Germany[16]	During the entire study period total: 336† LEA	NA	NA	NA	NA	NA	NA	NA	**IR**: DM pop.: reduced: RR per year 0.970 (0.943–0.997), p = 0.0318 non-DM pop.: unchanged: RR per year 1.029 (0.987–1.074), p = 0.186 **RRs**: decreased
Canavan et al., 2008, UK[38]	NA	1995–1996: 200.8 1996–1997: 117.2 1997–1998: 90.1 1998–1999: 177.1 1999–2000: 57.2	1995–1996: 7.3 1996–1997: 7.1 1997–1998: 9.6 1998–1999: 4.9 1999–2000: 11.0	NA	NA	1995–1996: 27.5† 1996–1997: 16.5† 1997–1998: 9.4† 1998–1999: 36.1† 1999–2000: 5.2† Estimation using quotient method§	NA	NA	**IR**: DM pop.: decreased descriptively# non-DM pop: increased descriptively# **RRs**: decreased descriptively
Icks et al., 2009, Germany[12]	During the entire study period total 427†LEA	48.0 (38.7–57.3)	9.5 (7.9–11.1)	NA	NA	5.1 (3.9–6.6) Estimation using quotient method§	Men: 5.3 (3.8–7.4) Women: 4.5 (3.1–6.6) Estimation using quotient method§	NA	NA
Ikonen et al., 2010, Finland[17]	Between 1997–2007 total 9,481; DM: 5,047 (53.2%) mean age 73.2 Y men: 69.8 Y women: 78.6 Y DD at LEA: 1997–2000 men: 14.4 Y women: 13.4 Y 2004–2007: men: 15.5 Y women: 14.6 Y non-DM: mean age 75.5 Y men: 70.2 Y women: 80.1 Y	1997: 94.4 2007: 48.3	1997: 10.7 2007: 8.0	NA	NA	1997–2007: 7.4 (7.2–7.7) Estimation using quotient method§	Men: 1997–2000: 11.7 (10.9–12.4) 2004–2007: 7.0 (6.5–7.4) 1997–2007: 8.9 (8.6–9.3) Women: 1997–2000: 8.8 (8.3–9.3) 2004–2007: 4.5 (4.2–4.8) 1997–2007: 6.3 (6.1–6.6)	NA	**IR**: in the DM. and non-DM. pop. significantly decreased **RRs**: significantly decreased
Lombardo et al, 2014, Italy[13]	Between 2003–2010 mean annual number of total LEA:4,954† DM: 2,435† (49,2%†) non-DM: 2,519†	2003: 48.4 2004: 44.1 2005: 42.8 2006: 41.8 2007: 36.1 2008: 36.8 2009: 33.2 2010: 36.1	2003: 5.3 2004: 5.0 2005: 4.7 2006: 4.6 2007: 4.5 2008: 4.7 2009: 4.4 2010: 4.2	RR men vs women: 2.0 (1.9–2.2)	NA	6.36 (5.6–7.23) Estimation using Poisson model	NA	NA	**IR**: DM pop.: decreased: RR per year 0.95 (0.94–0.97), p<0.001; non-DM pop. decreased: RR per year 0.98 (0.95–0.99), p<0.001 **RRs**: unchanged
*II*. *b*. *Incidence of major LEA*: *Hospital discharge rates*									
Wrobel et al., 2001, USA[41]	Between 1996–1997 total hospitalizations 83,710: DM: 44,599 (53.3%†) non-DM: 39,111	Nationwide:383 (360–406) 8.6-fold geografic variation	Nationwide: 38 (35.4–40.6) 6.7-fold geografic variation	NA	NA	10.1† Estimation using quotient method§	NA	NA	NA
Almaraz et al., 2012, Spain[3]	During the entire study period total hospitalizations: 9028† DM: 5858 (64.9%†) non-DM: 3170†	NA	NA	NA	NA	NA	NA	NA	**IR**: DM pop.: unchanged: RR per calendar year 1.00 (0.996–1.174), p = 0.263 non-DM pop: reduced: RR 0.982 (0.968–0.995), p = 0.007 **RRs**: increased descriptively
Buckley et al., 2012, Ireland[33]	During the entire study period hospitalizations DM: 585†	2005: 47.9 (37.8–59.5) 2009: 48.0 (37.3–60.4)	2005: 7.0 (6.0–8.2) 2009: 4.7 (3.9–5.6)	NA	NA	2005: 14.8 (11.8–18.6) 2006: 11.5 (9.0–14.7) 2007: 13.0 (10.3–16.3) 2008: 17.2 (13.6–21.7) 2009: 17.9 (13.9–23.0) Estimation using Poisson model	NA	NA	**IR**: DM pop.: unchanged, p = 0.23 non-DM pop.: dropped non significantly#, p = 0.16 **RRs**: descriptive
Lombardo et al., 2014, Italy[13]	Between 2003–2010 a mean annual number of total hospitalizations: 5,217† DM: 2,578† (49.4%†) non-DM: 2,639†	2003: 51.0 2004: 46.0 2005: 44.9 2006: 43.8 2007: 37.4 2008: 38.8 2009: 34.9 2010: 37.7	2003: 5.5 2004: 5.2 2005: 4.9 2006: 4.8 2007: 4.7 2008: 4.9 2009: 4.6 2010: 4.4	NA	NA	NA	NA	NA	**IR**: DM and non-DM pop.: decreased significantly **RRs**: unchange
*II*.*c*. *Incidence of major LEA*: *All LEAs per person*									
Calle-Pascual et al., 2001, Spain[15]	During the entire study period total LEA 455† DM: 267† (58.7%†)	NA	NA	Men: 1989–1993: 70.6 1994–1996: 41.4 1997–1999: 12.4 Women: 1989–1993: 15.3 1994–1996: 9.0 1997–1999: 5.6	Men: 1989–1993: 2.7 1994–1996: 1.1 1997–1999: 1.3 Women: 1989–1993:1.5 1994–1996: 0.4 1997–1999: 0.6	NA	NA	NA	**IR**: DM and non-DM pop.: decreased descriptively# **RRs**: NA
Canavan et al., 2008,UK[38]	NA	1995–1996: 310.5 1996–1997: 190.2 1997–1998: 132.9 1998–1999: 272.8 1999–2000: 75.8	1995–1996: 8.7 1996–1997: 9.6 1997–1998: 12.4 1998–1999: 8.1 1999–2000: 15.3	NA	NA	1995–1996: 35.5 (18.9–76.8) 1996–1997: 19.8† 1997–1998: 10.7† 1998–1999: 33.7† 1999–2000: 5.0 (2.82–9.43) Estimation using quotient method§	NA	NA	**IR**: DM pop.: decreased descriptively# non-DM pop: increased descriptively# **RRs**: decreased descriptively
**III. Incidence of minor LEA**									
*III*.*a*. *Incidence of minor LEA*: *One LEA per person*									
Morris et al., 1998, UK[39]	Between 1993–1994 total LEA: 87 DM: 23 (26.4%†) non-DM: 64	144.18	9.17	Men: 170.69 Women: 121.51	Men:9.73 Women:8.64	15.72 (9.58–25.80) Estimation using quotient method§	Men: 17.54 (10.98–28.03) Women: 14.06 (8.33–23.74) Estimation using quotient method§	NA	NA
Calle-Pascual et al., 2001, Spain[15]	NA	NA	NA	Men: 1989–1993: 52.1 (45.0–59.2) 1994–1996: 38.8 1997–1999: 22.5 (19.7–25.3) Women: 1989–1993: 10.9 (10.3–11.5) 1994–1996: 9.0 1997–1999: 7.9 (6.8–9.0)	Men: 1989–1993: 1.1# 1994–1996: 0.5 1997–1999: 0.7 (0.6–0.8) Women: 1989–1993:0.5 # 1994–1996: 0.1 1997–1999: 0.0	NA	NA	NA	**IR**: descriptive **RRs**: NA
Lombardo et al., 2014, Italy[13]	Between 2003–2010 mean annual number of total LEA: 6,406† DM: 4,355† (68%†) non-DM: 2,051†	2003: 95.7 2004: 102.6 2005: 95.1 2006: 93.7 2007: 94.6 2008: 85.5 2009: 91.6 2010: 89.4	2003: 3.4 2004: 3.4 2005: 3.7 2006: 4.0 2007: 3.8 2008: 3.9 2009: 3.9 2010: 4.0	RR men vs women: 2.6 (2.5–2.8)	NA	19.37 (16.49–22.77) Estimation using Poisson model	NA	NA	**IR**: DM pop.: unchanged: RR per calendar year 1.0 (0.99–1.01) p = 0.308 non-DM pop.: increased RR per calendar year 1.02 (1.01–1.03), p<0.01 **RRs**: unchanged
*III*.*b*. *Incidence of minor LEA*: *Hospital discharge rates*									
Almaraz et al., 2012, Spain[3]	Between 1998–2006 hospitalizations total: 7,007† DM: 5,742 (81.9%†) non-DM: 1,265	NA	NA	NA	NA	NA	NA	NA	**IR**: DM pop.: increased significantly: RR per calendar year 1.017 (1.007–1.027), p = 0.001 non-DM pop.: RR per calendar year 0.983 (0.677–1.004), p = 0.109 **RRs**: increased descriptively
Buckley et al., 2012, Ireland[33]	Between 2005–2009 hospitalizations DM: 1069†	2005: 96.2 (78.2–116.3) 2009: 127.6 (107.2–150.1)	2005: 5.0 (4.2–6.0) 2009: 4.5 (3.8–5.4)	NA	NA	2005:32.7 (26.2–40.9) 2006: 36.1 (28.6–45.6) 2007: 35.5 (28.5–44.1) 2008: 37.4 (29.8–46.9) 2009: 40.9 (33.0–50.7) Estimation using Poisson model	NA	NA	**IR**: DM pop.: increase non-significantly#, p = 0.11 non-DM pop.: unchanged, p = 0.55 **RRs**: descriptive
Lombardo et al., 2014, Italy[13]	Between 2003–2010 mean annual number of hospitalizations total: 7,934† DM: 5,576† (68%†) non-DM: 2,358†	2003: 120.1 2004: 127.9 2005: 120.2 2006: 116.7 2007: 117.3 2008: 107.1 2009: 113.1 2010: 110.8	2003: 4.0 2004: 4.0 2005: 4.3 2006: 4.5 2007: 4.3 2008: 4.4 2009: 4.5 2010: 4.5	NA	NA	NA	NA	NA	**IR**: DM Pop.: unchanged non-DM pop.: increased **RRs**: unchanged
*III*.*c*. *Incidence of minor LEA*: *All LEAs per person*									
Calle-Pascual et al., 2001, Spain[15]	NA	NA	NA	Men: 1989–1993: 58.9 1994–1996: 57.8 1997–1999: 33.1 Women: 1989–1993: 11.9 1994–1996: 11.3 1997–1999: 11.3	Men: 1989–1993: 1.4 1994–1996: 0.5 1997–1999: 0.7 Women: 1989–1993: 0.6 1994–1996: 0.1 1997–1999: 0.1	NA	NA	NA	**IR**: descriptive **RRs**: NA
Canavan et al., 2008, UK[38]	NA	1995–1996: 253.8 1998–1999: 362.9 1999–2000: 100.5	NA	NA	NA	NA	NA	NA	**IR**: DM pop.: unchanged# non-DM. pop.: NA **RRs**: NA

DPAR diabetic population at risk; LEA lower extremity amputation; AR attributable risk; PAR population attributable risk; DD diabetes duration; DM diabetes mellitus;

ITDM insulin-treated diabetes mellitus; NITDM non-insulin-treated diabetes mellitus; IR incidence rate; RR relative risks; Y year(s).

* Incidence rates per 100,000 person years.

^†^ self-calculated.

^§^ RR was calculated as quotient between incidence in the diabetic and non-diabetic populations.

^#^ Data as provided in the original paper.

**Fig 2:**
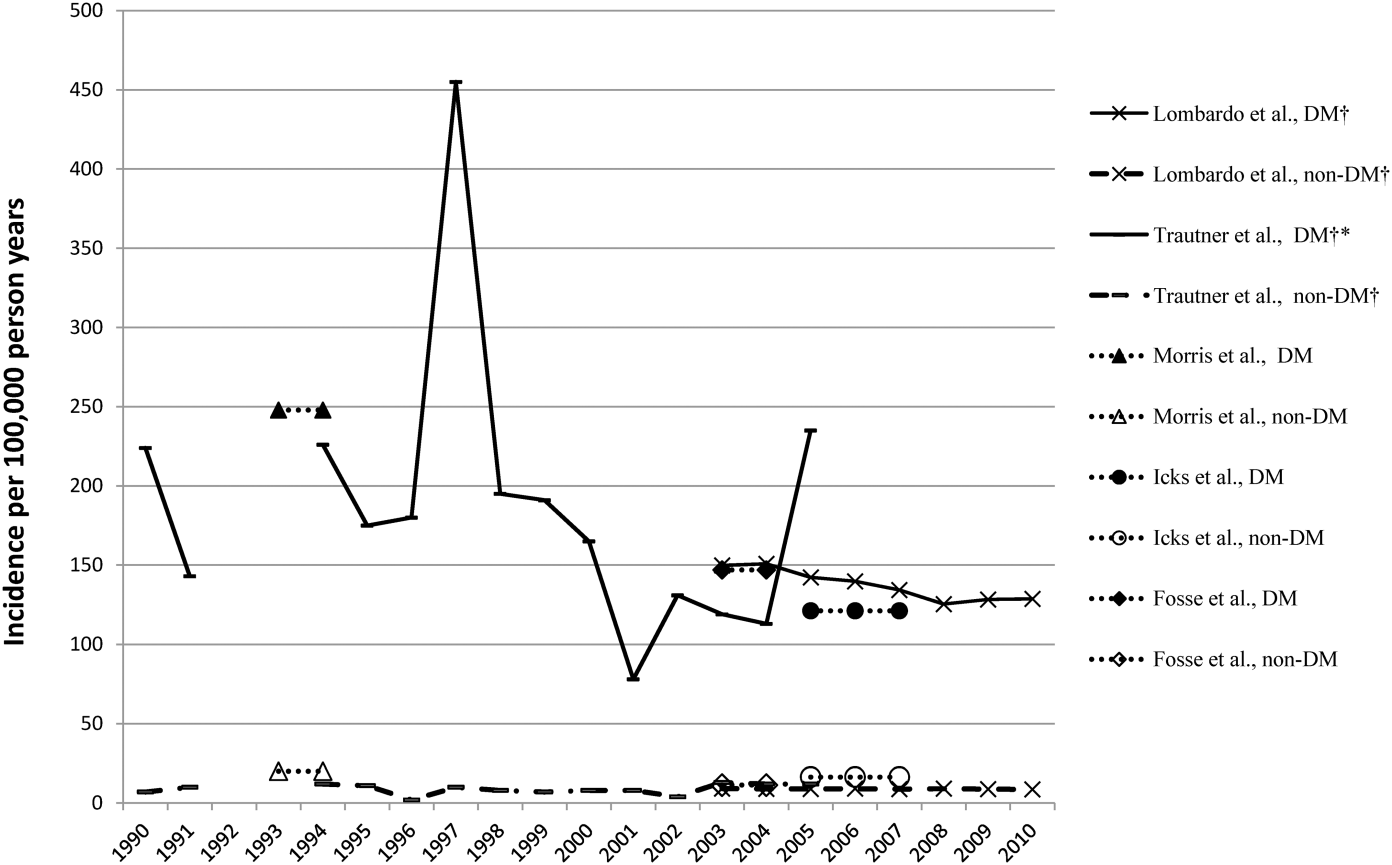
Incidence of any LEA (one LEA per person) in the diabetic compared with the non-diabetic population. † time trend was analysed; * significant time trend.

Incidence rates: The IRs ranged from 78 (95% CI 48–107) [16] to 455 (95% CI 0–989) [16] per 100,000 PY in the diabetic populations and from 2 (95% CI 0–5) [2] to 16.4 (95% CI 14.3–18.5) [12] in the non-diabetic population [12] (Table 2, Fig 2).

In both diabetic and non-diabetic populations, all studies found the highest IRs of LEAs among older patients. The majority of the studies described male gender as a risk factor for LEA with an approximately twofold increased incidence rate for men [2, 12, 13, 37].

Ethnic differences: Gujral et al. reported lower IRs among patients of Asian ethnicity compared with white Caucasians [40].

**Fig 3:**
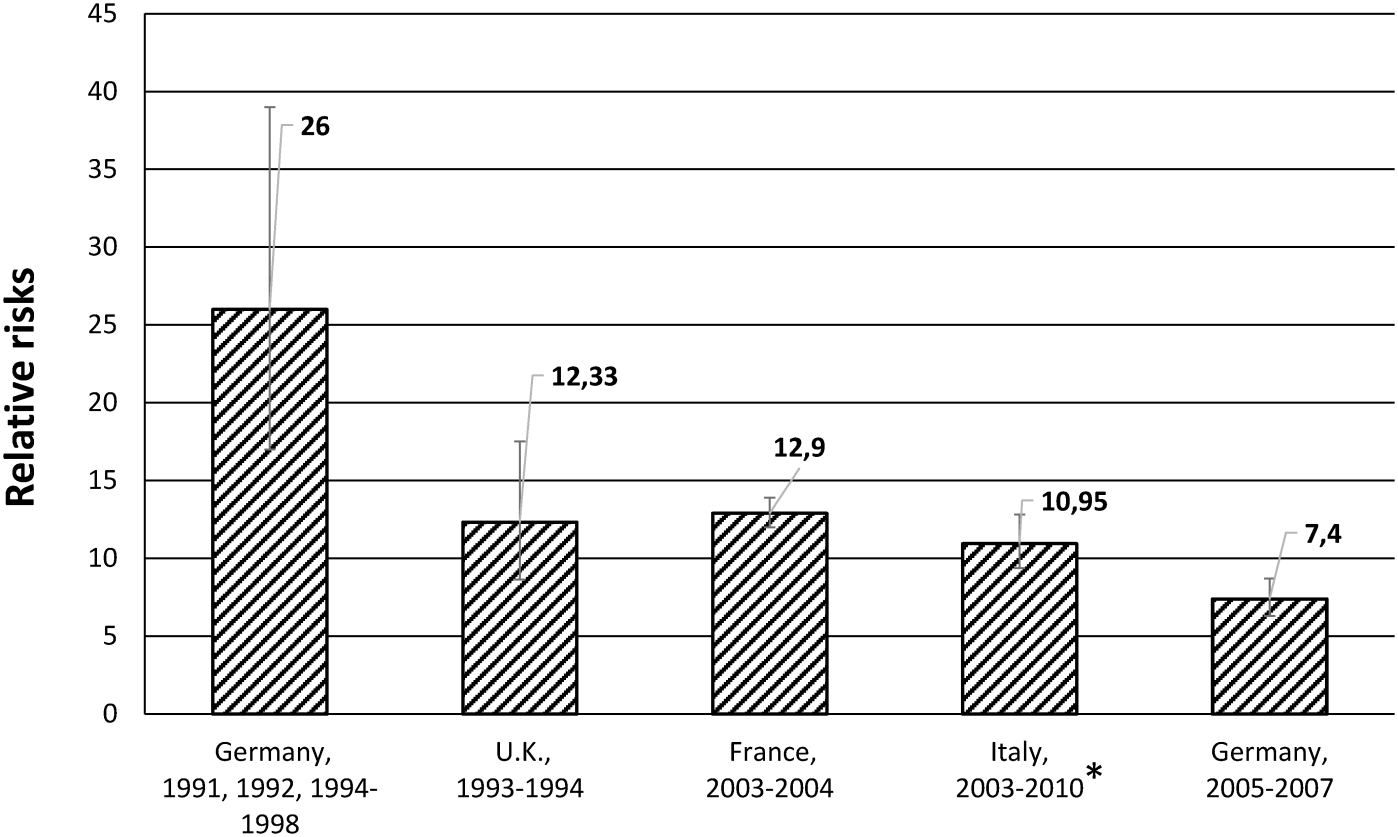
Relative risks of any LEA (one LEA per person) in the diabetic compared with the non-diabetic population. * RRs from Poisson model; other studies as quotients from IRs.

Relative, attributable and population attributable risks between the diabetic and non-diabetic population: The RRs ranged from 7.4 (95% CI 6.3–8.7) [12] to 26 (95% CI 17–39) [2] (Table 2, Fig 3). The RRs in diabetic compared with non-diabetic persons decreased with increasing age [12, 39]. Two studies reported higher RRs among men [12, 39] and one among women [37]. Based on the studies available it was not possible to estimate the ethnic differences regarding the RRs.

The attributable risk among persons with diabetes varied from 86% [12] to 99% [16] and the population-attributable risk ranged from 26% [39] to 90% [16].

Time trends of the incidence rates: Out of three publications that analysed the time trend [2, 13, 16], only one study [16] was able to find a significant reduction in IRs of LEAs in the diabetic population: RR per calendar year 0.976 (95% CI 0.958–0.996), whereas in the non-diabetic population the secular trend was unchanged (Table 2, Fig 2).

Time trends of the relative risks: The study by Lombardo et al. [13] found no change in RRs whereas the study by Trautner et al. [16] found a significant reduction of RRs during the study period.

#### I.b. Incidence of any LEA: Hospital discharge rates

Eight studies reported the incidence of any LEA based on discharge data [3, 11, 13, 19, 33–36]. The proportion of diabetes-related LEA ranged from 47% [35, 36] to 72.6% [3] (Table 1).

**Fig 4:**
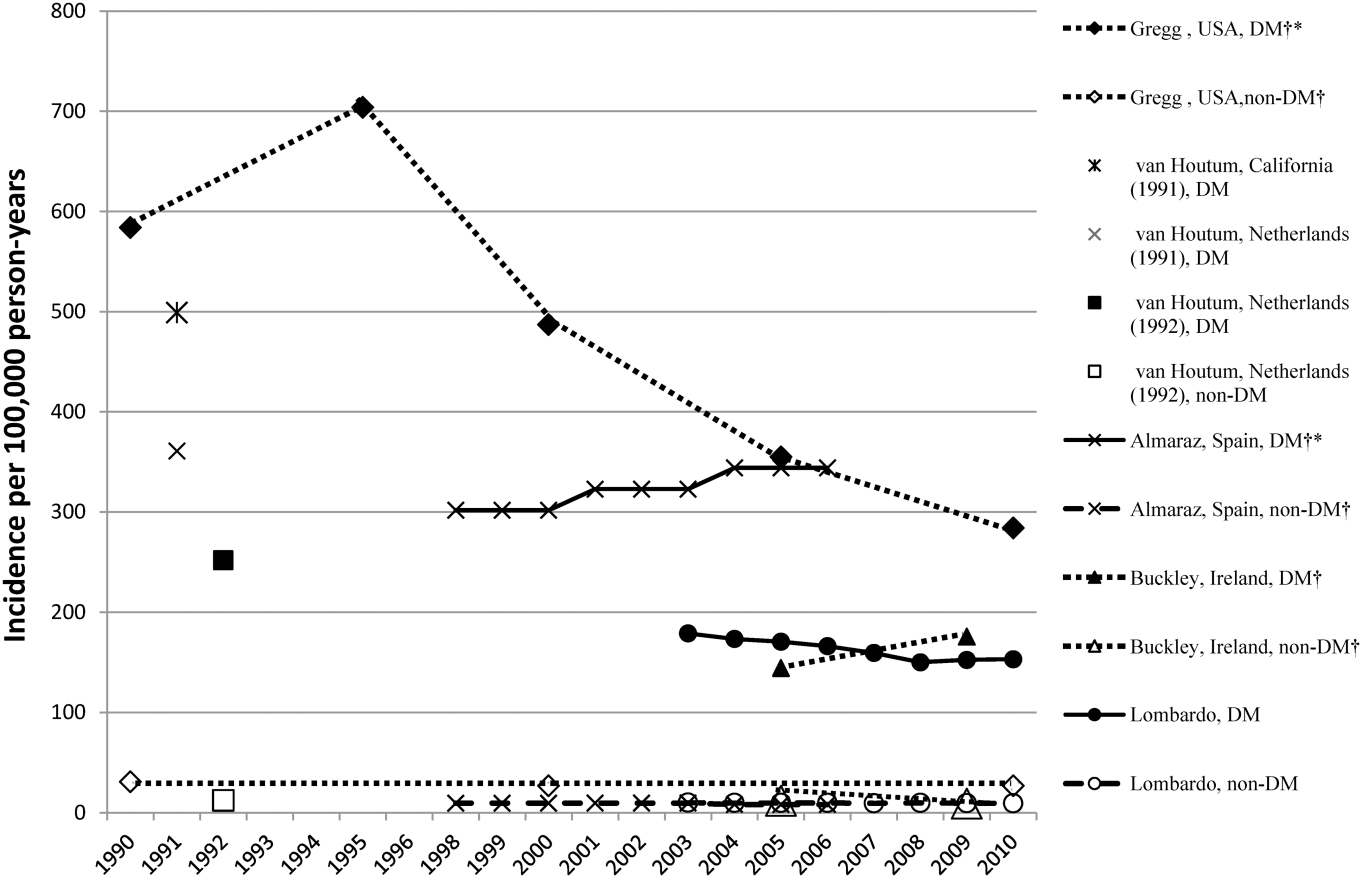
Incidence of any LEA (hospital discharge rates) in the diabetic compared with the non-diabetic population. † time trend was analysed; * significant time trend.

Incidence rates: In the diabetic population IRs (per 100,000 PY) varied from 144 (95% CI 123.2–166.9) in Ireland, 2005 [33] to 704 (95% CI 591–817) in the USA, 1995 [19]. In the non-diabetic population IRs varied from 8.3 (95% CI 7.6–9.1) in Spain, 2004–2006 [3] to 31 (95% CI 27–35) in the USA, 1990 [19] (Table 2, Fig 4).

With respect to age and gender differences, this group of studies showed similar patterns to studies that analysed one LEA per person. One study reported higher IRs among African-Americans [11]. The available studies showed higher IRs of LEAs in the USA than in Europe until the early 2000s [19,34], and the study by Van Houtum also identified regional differences [35].

**Fig 5:**
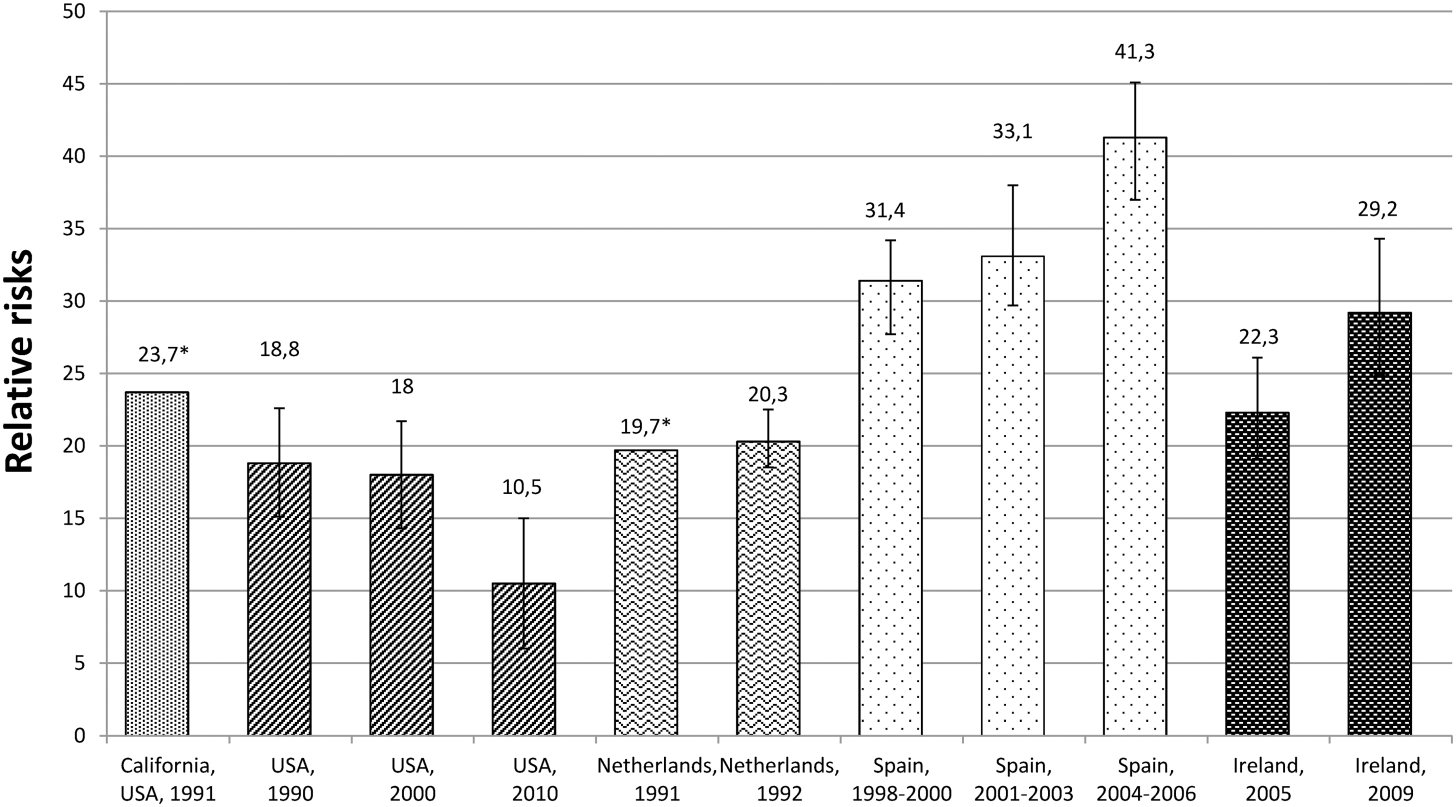
Relative risks of any LEA (hospital discharge rates) in the diabetic compared with the non-diabetic population. * 95% Confidence interval was not reported.

Relative risks between diabetic and non-diabetic populations: The RRs varied between 10.5 in the USA, 2010 (95% CI 6.0–15.0) [19] and 41.3 in Spain, 2004–2006 (95% CI 37.0–45.1) [3] (Table 2, Fig 5).

Two studies [3, 36] reported the reduction of RRs between diabetic and non-diabetic populations with increasing age. Concerning gender differences, the study by Almaraz et al. [3] described the higher RRs between 1998–2003 among women and between 2004–2006 among men, whereas the study by Van Houtum et al. found no differences [35]. Interestingly, one US study which presented data stratified by ethnic origin found lower RRs among African-American persons than among non-Hispanic whites and Hispanic persons [11]. One study from the 1990s reported higher RRs in the USA than in Europe [34]. In contrast, since the 2000s the reported RRs were higher in Europe [3, 33] than in the USA [19].

Time trends of the incidence rates: Three studies described the time trend in the diabetic population with contradictory results [3, 19, 33]. The study by Gregg et al. [19] reported a significant reduction of IRs between 1990–2010 in the USA. In contrast, the study by Almaraz et al. [3] reported a significant increase of IRs between 1998–2006 in Andalusia, Spain, while a study by Buckley et al. [33] showed non-significant growth of IRs in Ireland between 2005–2009. All studies found decreased IRs in the non-diabetic population over time.

Time trends of the relative risks: the study by Gregg et al. [19] reported a substantial decrease of RRs over time, while the study by Almaraz [3] described the increasing RRs during the study period and the study by Buckley et al. found no significant changes [33].

#### I.c. Incidence of any LEA: All LEAs per person

Only one study in the UK described IRs regarding all LEAs per person [38]. This study reported a reduction of both IRs in the diabetic population from 564.3 to 176.0 per 100,000 PY as well as RRs from 46 (95% CI 25.7–90.6) to 7.7 (95% CI 4.99–12.9) between 1995–1996 and 1999–2000 [38] (Table 2).

#### II. Incidence of major LEA

Eight studies analysed the IRs of major LEAs counting one amputation per person [2, 12, 13, 15–17, 38, 39], four studies estimated the incidence of major LEA based on the hospital discharge rates [3, 13, 33, 41], and two described all major LEAs per person [15, 38]. The proportion of LEAs among persons with diabetes from all LEAs ranged from 27.6% [39] to 64.9% [3] (Table 1).

Incidence rates: The IRs of major LEA among persons with diabetes varied from 33.2 (95% CI NA) per 100,000 PY in Italy, 2009 [13] to 383 (95% CI 360–406) per 100,000 PY among persons aged ≥ 65 years in the USA, 1996–1997 [41] (Table 2, S1 Fig). Among persons without diabetes the IRs of major LEA ranged from 4.2 (95% CI NA) per 100,000 PY in Italy, 2010 [13] to 38 (95% CI 35.4–40.6) among persons aged ≥ 65 years in the USA, 1996–1997 [41] (Table 2). Men were more likely to undergo major LEAs than women [12, 13, 15, 39]. Large regional differences in IRs of major LEA were shown in the study from the USA [41].

Relative risks between diabetic and non-diabetic population: Relative risks of major LEAs lie between 5.1 in Germany, 2005–2007 (95% CI 3.9–6.6) [12] and 35.5 (95% CI 18.9–76.8) in the UK, 1995–1996 [38]. RRs were consistently higher among men than women [12, 17, 39].

Time trends of the incidence rates: Among the diabetic population only those studies which estimated one major amputation per person found a significant reduction of IRs of major LEAs during the study period (S1 Fig) [13, 16, 17, 38]. Among the non-diabetic population some studies also reported decreased secular trends [3, 13, 17].

Time trends of the relative risks: Among the studies that counted one major LEA per person and analysed a time trend, two found significantly decreased RRs over time [16, 17] whereas one found no change in RRs [13]. Another study counting all major LEAs also showed a descriptive reduction during a 5 year period from 35.5 (95% CI 18.9–76.8) to 5.0 (95% CI 2.82–9.43) respectively [38].

#### III. Incidence of minor LEA

Three studies estimated IRs of minor LEA counting one LEA per person [13, 15, 39], three studies were based on hospitalization rates [3, 13, 33], and two described all minor LEAs per person [15, 38].

Incidence rates: As expected, the highest IRs of minor LEA in the diabetic population were found in the group counting all LEAs per person with a maximum 362.9 (95% CI NA) per 100,000 PY in the UK, 1998–1999 [38], and the lowest IRs of 85.5 (95% CI NA) per 100,000 PY in Italy, 2008 [13] were described in the group counting one LEA per person. In the non-diabetic population IRs were not higher than 10 per 100,000 PY and were reasonably comparable [13, 15, 33]. Two studies found higher IRs of minor LEAs among men than women [15, 39].

Relative risks between diabetic and non-diabetic populations: RRs between diabetic and non-diabetic populations were generally higher than those for major LEAs and ranged between 15.72 (95% CI NA) in 1993–1994 in UK [39] and 40.9 (95% CI 33.0–50.7) in 2009 in Ireland [33].

Time trends of the incidence rates: Among persons with diabetes one study found no significant change of IRs of minor LEA over time [13], while others showed a significant [3] and a non-significant increase [33] of IRs. Among persons without diabetes one study described increased [13] and two studies unchanged secular trends [3, 33].

Time trends of the relative risks: Only the study by Lombardo et al. analysed a time trend of RRs and found no significant changes during the study period [13].

## Discussion

This systematic review, comprising 19 studies, demonstrates considerable variations in incidence of LEA as well as in relative risks of amputations in the diabetic compared with the non-diabetic population. This variation may probably be due to the large heterogeneity of the studies included.

### Main findings

In 1989, the St. Vincent Declaration set the goal to reduce the incidence of LEA by half within five years [9] and thus to approach the incidence in the non-diabetic population. Still there remains uncertainty as to whether this goal has been achieved. The time trend of both incidence rates and relative risks in the diabetic as well in the non-diabetic population varied with different definitions of LEA (any, minor, major) and methods of recording (one LEA per person or more). The previous reviews [25, 27] already highlighted the importance to take into account the methodological discrepancies between studies by interpreting the published data.

#### Incidence rates of LEA

The studies reviewed showed considerable variation in incidence rates of LEA in both the diabetic and non-diabetic populations. Nevertheless, there were some patterns that can be described.

Gender differences: Some studies found higher IRs among men than women in both the diabetic and the non-diabetic population. The gender-relative risks ranged from 1.5 [37] to 3.0 [3] for any LEAs and from 1.56 [39] to 5.0 [15] for major LEAs. Moreover, men were younger at the time of LEA then women [2, 3, 12, 16, 17, 39]. This strong association between risk of LEAs and male gender was described in earlier publications [47]. The increased IRs among men may be explained by environmental factors such as smoking as well as by higher prevalence of peripheral vascular disease, peripheral neuropathy and diabetic foot ulceration [35, 47], but not by healthcare factors [47].

Ethnic differences: Two studies, both from the USA, found higher IRs among African-Americans than white Caucasians in both the diabetic and non-diabetic populations [11, 19]. Risk factors such as smoking, low socio-economic status, and poor access to healthcare may contribute to observed ethnic disparities [27, 48]. Moreover, the African-American ethnicity *per se* could be an independent risk factor for LEA [27, 49]. Nevertheless, it is probably true only for the African-American ethnicity in the USA. One recent study by Holman found no significant differences in the crude incidence rates of LEA between black and white residents in England [21]. The reason for these contrasting findings could be due to the organization of the healthcare systems: private in the USA and a public healthcare system in the UK. Interestingly, that one reviewed study demonstrated a lower incidence of LEA in Asian diabetic patients than in white patients [40]. This finding is in line with the study by Holman mentioned above [21]. The lower prevalence of PAD [50] or neuropathy [51] among Asians may be possible protective factors.

International and regional differences: An international comparison showed a higher incidence of LEA in both diabetic and non-diabetic populations in the USA than in Europe until the early 2000s [19, 34]; in later years the incidences were converging [19]. Regional disparities within a country were found in both the USA [41] and Europe [35], being more pronounced in the diabetic than in the non-diabetic population [35, 41]. The RRs between diabetic and non-diabetic populations varied widely across regions, too [35, 41]. Differences in the regional distribution of Asian or Black ethnicity [21, 52], differences in access to healthcare [41, 52] as well as in the organization of diabetes care and the quality of foot-care centers [21, 41, 52] were described as potential influencing factors.

Time trends: Only studies that analysed the incidence rates of one major LEA per person found a significant decrease in the incidence of LEAs in the diabetic population over time (Table 2). No clear trends in the incidence of LEA could be observed in studies that analysed more than one major LEA or in studies that analysed minor LEA irrespective of the counting methods (Table 2). For studies among the non-diabetic population no time trend could be observed (Table 2). We consider it important to analyse the time trend of LEAs depending on the extent of amputations, as the clinical distinction and objectives of performing major and minor LEAs are different. The reduction regarding incidence of major LEA in the diabetic population could be explained in particular by better organised multidisciplinary care for patients with diabetic foot [13, 16, 38] but also by improvements in diabetes care [13, 15, 19], tighter control of hypertension and hypercholesterolemia [15], and reduced tobacco and alcohol consumption among persons with diabetes [15]. Improvements in bypass surgery as well as new endovascular revascularization techniques are important factors for the reduction of LEA incidence among patients with peripheral vascular disease [53–56]. However, not all reviewed studies showed significant reduction of LEA incidence in the non-diabetic population. The reduced incidence of major LEA can therefore be explained only in part by the success of vascular surgery [57].

#### Relative risks between the diabetic and non-diabetic populations

In general, RRs between diabetic and non-diabetic persons for minor LEAs were more than twice as high as for major LEAs [13, 33]. Similarly, RRs based on hospital discharge rates were higher than in studies counting one LEA per person [13]. Some studies reviewed reported decreased RRs with increased age of patients at the time of LEA [3, 12, 36, 39]. This could be explained by the substantial growth in IRs of LEAs due to other reasons among older non-diabetic patients when compared with diabetic patients from the same age group. Regarding gender differences, the results were not fully consistent: most studies reported higher RRs among men [3, 12, 17, 39], one among women [3, 37] and another described similar RRs [35]. The RRs among African-Americans were lower than among non-Hispanic whites and Hispanics [11]. Until the 2000s the RRs were higher in the USA than in Europe [34], although that trend seems now to have reversed [3, 19, 33]. Most studies that analysed one major LEA showed that the relative risks among persons with diabetes has been decreasing over time compared with persons without diabetes [16, 17, 38]. The results on time trends of RRs of LEA in the other groups of studies were contradictory (Table 2).

### Risk of bias within studies

Selection bias regarding the study population was minimized through the restriction to population-based studies. At the same time, some sources for information bias were detected.

Firstly, it appears reasonable to assume that the registration of major LEAs in hospitals is complete (owing to the fact that hospital reimbursement often depends on the major amputation procedures). However, the number of minor LEAs based on the hospital data could be underestimated due to incomplete documentation, since documenting more than one minor LEA during one hospital stay does not normally trigger higher refunds and may therefore be neglected. Also, autoamputations or minor LEAs performed outside the operating theatre are presumably underreported.

Secondly, in most studies the definition of diabetes among amputees was based on the coding of diabetes diagnosis in admission or discharge records. However, it was shown that diabetes diagnosis based on hospital admissions data can lead to underreporting of diabetes by up to 15% [13, 18, 58] and thus to a considerable underestimation of the incidence of LEAs in the diabetic population.

Thirdly, most studies used survey data for the estimation of a “diabetic population at risk” or created a special algorithm to identify persons with diabetes. However, these methods could lead to some misclassification, in particular underestimation when a diabetes diagnosis is not documented [12, 16]. Furthermore, patients with undiagnosed diabetes might have been misclassified as non-diabetic patients. Only in one study by Calle-Pascual were patients with undiagnosed diabetes also taken into account [15]. Most studies also used constant diabetes prevalence during the study period. In this case, instant diabetes prevalence is underestimated [2, 3, 16]. Moreover, the change in 1997 in the diagnostic criteria for diabetes from 140 mg/dl (7.8 mmol/l) to 126 mg/dl (7.0 mmol/l) fasting plasma glucose [59] led to an increase of the diabetic population due to the inclusion of less severe stages of the disease, and this must also be taken into consideration when interpreting the results.

Fourthly, the vast majority of the studies reviewed were not able to distinguish between type 1 and type 2 diabetes. It is therefore not clear if the incidence and the time trend of LEAs among patients with type 1 diabetes differ from those among patients with type 2 diabetes.

Finally, low absolute numbers of LEAs in small study populations may cause strong variations in the incidence rates [2, 16]. This complicates the appropriate interpretation of the data.

### Risk of bias across studies

Due to the fact that only articles published in the English language were reviewed, publication (language) bias could not be ruled out. Although we searched five databases, we cannot guarantee that some related papers may not have been identified. However, we did check the reference lists of reviewed articles to identify relevant studies.

Furthermore, differences in methodological approaches across studies can lead to limited comparability. The studies reviewed used different definitions of major amputations: through or proximal to the ankle joint [39], any LEA above the ankle joint [2, 13, 16, 41]; any LEA proximal to the midtarsal level (International Consensus of the Diabetic Foot) [12]; through or proximal to the tarsometatarsal joint (GLEAS protocol) [15, 38]. However, hindfoot amputations such as Syme’s, Chopart or Lisfranc may constitute a relevant proportion of all amputations [60]. For the comparability of future studies it is therefore clearly important to find an unequivocal differentiation between major and minor LEA, since the percentage of amputations “in between” is not marginal.

Differences were also observed in the way the studies reviewed reported the causes of LEAs. Most studies analysed non-traumatic LEAs, three estimated incidence of all LEAs regardless of their cause [12,17,18], a number analysed “diabetes-related” (not traumatic and not tumour-related) LEAs [3, 13, 39, 40] and one study from Finland analysed only LEAs attributable to peripheral vascular disease [37]. However, it was shown that traumatic LEAs are less frequent in diabetic than in non-diabetic populations (6.5% vs. 15%) [18].

With regard to the divergent definition of diabetic population at risk, only few studies used data based on actual registers, which means almost complete data collection [17, 37]. In contrast, most studies used the estimated prevalence of diabetes. Moreover, most studies reviewed the estimated diabetic population at risk as a percentage of patients with known diabetes, one study as a percentage of patients with known and unknown diabetes [15], and two used the denominator based on the number of patients with treated diabetes [17, 18]. Due to the high prevalence of unknown type 2 diabetes [1, 61, 62], it is expected that the incidence of LEAs with the denominator based on known and unknown diabetes will be considerably lower than where the denominator is based on a population with known diabetes. Where the denominator is based on drug-treated diabetes it should be taken into account that the percentage of diet-treated type 2 diabetes patients could amount to up to 25% [63].

Finally, specific characteristics of the study population such as age or ethnicity could also influence the results. Most studies had no age restriction, but some studies analysed the population aged ≥20 years [33] or aged ≥30 years [3, 19], and the study by Wrobel et al. [41] analysed the Medicare population aged ≥65 years. Study populations from the USA [11, 19, 34, 41] are clearly distinguished from European studies by a high proportion of African-American and Hispanic persons.

### Strengths and limitations

The selection of studies for this systematic review was based on a systematic search approach with clearly determined search strategies. Two independent reviewers screened the articles and performed the data extraction. We included only those studies reporting IRs of LEA within the population at risk, i.e. the diabetic population. The advantage of this method over IRs of LEA within the general population is that the results are not influenced by changes in the prevalence of diabetes. Moreover, we analysed incidences of LEA in the diabetic population in separate groups according to definition of LEA as well as the method of recording and study design (study characteristics). This approach allows comparison of the studies despite a high degree of heterogeneity. Nevertheless our review has some limitations. Although seven databases were searched, we cannot rule out having missed any relevant studies due to publication bias. Furthermore, studies that were published in languages other than English were excluded. Most studies reporting on IRs of LEA among patients with diabetes within the diabetic population were conducted in economically developed areas such as the USA and Europe, and thus do not represent a worldwide perspective.

## Conclusion

This comprehensive review demonstrates the considerable variation in incidence of LEA among the diabetic population, probably partly due to a large heterogeneity of identified studies. As expected, the incidence of LEA was higher in the diabetic than in the non-diabetic population. Most studies found a higher incidence of LEA among male diabetic patients. Black and Hispanic patients have a higher risk of LEA than white individuals, Asian patients, however, do not. Studies that analysed one major LEA found decreased incidence rates among the diabetic population as well as corresponding relative risks over time. Among studies with different study design, the current data on time trends for incidence rates as well as relative risks between diabetic and non-diabetic populations are contradictory. The studies reviewed showed high regional and international differences with respect to both incidence and relative risks of LEA. A comparison was difficult due to the lack of consensus between the studies’ methods. We recommend that future studies analysing the incidence and relative risks of LEA in the diabetic population should use a comparable study design regarding anatomic definition, cause and recording of LEAs as well as the population at risk.

## Supporting information

S1 Table PRISMA 2009 checklist. (DOC)

S2 Table Methodology checklist. Critical appraisal.
(PDF)

S1 Text PROSPERO registration number. (PDF)

S2 Text Search strategies. (DOCX)

S1 Fig Incidence of major LEA (one LEA per person) in the diabetic compared with the non-diabetic population. (TIFF)
