# Supplementary material for: Incidence of lower extremity amputations in the diabetic compared with the non-diabetic population: A systematic review
Source: PLoS One. 2017 Aug 28;12(8):e0182081. doi: 10.1371/journal.pone.0182081 (PMC5573217; doi:10.1371/journal.pone.0182081)
Supplement: S2 Table — Critical appraisal. (PDF) [file pone.0182081.s002.pdf]

Methodology Checklist Critical appraisal modified by Methodological Evaluation of Observational Research[22] (MORE, Shamliyan TA, 2013) and the Scottish Intercollegiate Guidelines Network[23] (SIGN, http://www.sign.ac.uk/methodology/checklists.html)

**Project:** Incidence of lower-limb amputations in the diabetic compared to the non-diabetic population: A Systematic Review			
Study (*author, title, year of publication, journal title, pages*)			
**Section 1: Internal validity**			*Does this study do it?*
1	The incidence of amputations is a main question of the study	Yes □	No □
**Population**			
2	Precise description of the method of estimating the population at risk (diabetic population)?	Yes □	No □
3	Precise description of the source of data (survey, diabetes registry, insurance data…)	Yes □	No □
4	Was incident diabetes taken into account?(only for diab./non-diab. population)	Yes □	No □
5	Precise description of the method of diagnosis of diabetes among patients with amputations?	Yes □	No □
6	Precise description of the source diagnosis of diabetes (self-reported, physician DS (ICD), insurance data)	Yes □	No □
**Outcome LEAs**			
7	Appropriate clinical definition of LEA e.g. re-amputations not appropriate, only tumor or trauma indication clearly excluded…)	Yes □	No □
8	Clear description of the definition of anatomical level (minor/ major/total amputation)	Yes □	No □
9	Precise description of the source for the estimation of amputations: hospital discharge, OPS…	Yes □	No □
10	Were the estimates for all parameters (IR, RR) with 95% confidence interval reported?	Yes □	No □
11	Precise description of calculation of incidence rates concerning study and reference population (reference population /denominator)?	Yes □	No □
12	Time trend reporting	Yes □	No □
13	Time trends reported using appropriate multivariate regression model	Yes □	No □
14	Absolute numbers of cases reported	Yes □	No □
15	No. of amputations is not below 10	Yes □	No □
16	The potential sources of limitations and bias were mentioned	Yes □	No □
**Section 2: OVERALL ASSESSMENT OF THE STUDY**			
	Overall assessment of the methodological quality of this study	High quality (++) □<br>Acceptable (+) □<br>Low quality (-) □	
High quality (++): Majority (13 points or more) of criteria met. Little or no risk of bias. Acceptable (+): Most (8 points or more) of criteria met. Some flaws in the study with an associated risk of bias. Low quality (-): Either most criteria not met, or significant flaws relating to key aspects of study design.			
