# Supplementary material for: Incidence of lower extremity amputations in the diabetic compared with the non-diabetic population: A systematic review
Source: PLoS One. 2017 Aug 28;12(8):e0182081. doi: 10.1371/journal.pone.0182081 (PMC5573217; doi:10.1371/journal.pone.0182081)
Supplement: S1 Text — (PDF) [file pone.0182081.s003.pdf]

Dear Mrs Kvitkina

Thank you for submitting details of your systematic review *Incidence of lower-limb amputations in the diabetic compared to the non-diabetic population: a systematic review protocol* to the PROSPERO register. We are pleased to confirm that the record has been published on the database.

Your registration number is: CRD42015017809

You are free to update the record at any time, all submitted changes will be displayed as the latest version with previous versions available to public view. Please also give brief details of the key changes in the Revision notes facility. You can log in to PROSPERO and access your records at http://www.crd.york.ac.uk/PROSPERO

An email reminder will be sent to you on the anticipated completion date, prompting you to update the record.

Comments and feedback on your experience of registering with PROSPERO are welcome at crd-register@york.ac.uk

Best wishes for the successful completion of your review.

Yours sincerely

Jimmy Christie

PROSPERO Administrator
Centre for Reviews and Dissemination
University of York
York YO10 5DD
t: +44 (0) 1904 321040
f: +44 (0) 1904 321041
e: CRD-register@york.ac.uk
www.york.ac.uk/inst/crd

CRD is part of the National Institute for Health Research and is a department of the University of York.

Email disclaimer: http://www.york.ac.uk/docs/disclaimer/email.htm
