# Supplementary material for: Incidence of lower extremity amputations in the diabetic compared with the non-diabetic population: A systematic review
Source: PLoS One. 2017 Aug 28;12(8):e0182081. doi: 10.1371/journal.pone.0182081 (PMC5573217; doi:10.1371/journal.pone.0182081)
Supplement: S2 Text — (DOCX) [file pone.0182081.s004.docx]

# Search strategies

Report of search strategies as conducted in January 2013. All strategies have been updated in December 2014.

#### 1 MEDLINE

Database: MEDLINE (PubMed, NLM)

Search period: unlimited

Date of search: 28.01.2013

|  |  |  |
| --- | --- | --- |

| **Step** | **Hits** | **Search** |
| --- | --- | --- |
| #1 | 15525 | "AMPUTATION"[MESH] |
| #2 | 28894 | disarticulation [tiab] OR amputat* [tiab] OR amputee [tiab] |
| #3 | 34921 | #1 OR #2 |
| #4 | 189074 | "Hospitals"[Mesh] |
| #5 | 19776 | "EPIDEMIOLOGY"[MESH] |
| #6 | 470086 | epidemiolog* [ti] OR prevalence [ti] OR incidence [ti] OR frequency [ti] OR population survey [tiab] OR survey data [tiab] OR administrat* data [tiab] OR occurence [ti] OR morbidity [ti] OR mortality [ti] OR population data [tiab] OR population-based [tiab] OR community data [tiab] OR community-based [tiab] |
| #7 | 488141 | #5 OR #6 |
| #8 | 811 | #3 AND #7 |
| #9 | 56 | #8 AND (hospital [ti] OR inpatient [ti] OR hospitali* [ti] OR department [ti]) |
| #10 | 45 | #8 AND #4 |
| #11 | 83 | #9 OR #10 |
| #12 | 751 | #8 NOT #11 |
| #13 | 1 | 21388445 [PMID] |
| #14 | 121 | Related Citations for PubMed (Select 21388445) |
| #15 | 1 | 16779516 [PMID] |
| #16 | 162 | Related Citations for PubMed (Select 16779516) |
| #17 | 168 | (epidemiology [ti] OR epidemiologic [ti] OR epidemiological [ti] OR prevalence [ti] OR incidence [ti]) AND (foot [ti] OR feet [ti] OR toe [ti] OR limb [ti] OR limbs [ti] OR extremity [ti] OR extremities [ti]) AND (disarticulation [tiab] OR amputat* [tiab] OR amputee [tiab]) |
| #18 | 189 | (epidemiology [ti] OR epidemiologic [ti] OR epidemiological [ti] OR prevalence [ti] OR incidence [ti]) AND (disarticulation* [ti] OR separation* [ti] OR amputat* [ti] OR hemipelvectom* [ti] OR amputee [ti]) |
| #19 | 1132 | #12 OR #14 OR #16 OR #17 OR #18 |

#### 2 EMBASE, Journals@OVID

Database: MEDLINE (1946-2012), EMBASE (1974-2012), Journals@OVID (OVID)

Search period: unlimited

Date of search: 28.01.2013

| **Step** | **Hits** | **Search** |
| --- | --- | --- |
| 1 | 11695 | *AMPUTATION/ |
| 2 | 22421 | (disarticulation OR amputat* OR amputee).m_titl. |
| 3 | 26277 | 1 OR 2 |
| 4 | 115008 | *LOWER EXTREMITY/ OR *FOOT/ OR *HIP/ OR *KNEE/ OR *LEG/ OR *THIGH/ |
| 5 | 295779 | (limb* OR foot OR feet OR extrem* OR leg OR toe).m_titl. |
| 6 | 367975 | 4 OR 5 |
| 7 | 10687 | 3 AND 6 |
| 8 | 43536 | *EPIDEMIOLOGY/ |
| 9 | 1013909 | (epidemiolog* OR prevalence OR incidence OR frequency OR population survey OR survey data OR hospital data OR administrat* data OR occurence OR morbidity OR mortality OR population data OR community data OR community-based OR population-based).m_titl. |
| 10 | 1038241 | 8 OR 9 |
| 11 | 521 | 7 AND 10 |
| 12 | 272 | ((epidemiology OR epidemiologic OR epidemiological OR prevalence OR incidence) AND (foot OR feet OR toe OR limb OR limbs OR extremity OR extremities) AND (disarticulation OR amputat* OR amputee)).m_titl. |
| 13 | 429 | ((epidemiology OR epidemiologic OR epidemiological OR prevalence OR incidence) AND (disarticulation* OR amputat* OR amputee)).ti. |
| 14 | 652 | 11 OR 12 OR 13 |
| 15 | 370 | remove duplicates from 14 MEDLINE: 49 EMBASE: 267 Journals@Ovid: 54 |

#### 3 Web of Knowledge

Database: Web of Knowledge (Thomson Reuters)

Search period: unlimited

Date of search: 28.01.2013

| **Step** | **Hits** | **Search** |
| --- | --- | --- |
| 1 | 350 | Title=((amputat* OR disarticulation OR amputee)) AND Title=((epidemiolog* OR prevalence OR incidence OR morbidity OR mortality)) Timespan=All Years Lemmatization=On |

#### 4 ScienceDirect

Database: ScienceDirect (Elsevier, Segmente “Medicine and Dentistry”, “Neuroscience”, “Nursing and Health professions”)

Search period: unlimited

Date of search: 28.01.2013

| **Step** | **Hits** | **Search** |
| --- | --- | --- |
| 1 | 14 | TITLE((amputat* OR disarticulation OR amputee)) AND TITLE-ABSTR-KEY((epidemiolog* OR prevalence OR incidence OR frequency OR population survey OR survey data OR administrat* data OR occurrence OR morbidity OR mortality OR population data OR population-based OR community-based OR community data)) |

#### 4.5 CCMed, Deutsches Ärzteblatt

Database: CCMed, Deutsches Ärzteblatt (DIMDI)

Search period: unlimited

Date of search: 28.01.2013

| **Step** | **Hits** | **Search** |
| --- | --- | --- |
| 1 | 54 | (FT=(amputat? ; disarticulation ; amputee ) AND FT=(epidemiolog? ; prevalence ; incidence ; frequency ; population survey ; survey data ; administrat? data ; occurence ; morbidity ; mortality ; population data ; community data ; population-based ; community-based) |

Update: Time period up to 19^th^ of January December 2014

#### 1 MEDLINE a

Database: MEDLINE (PubMed, NLM)

Search period: 2013-2014

Date of search: 19.12.2014

| **Step** | **Hits** | **Search** |
| --- | --- | --- |
| #1 | 16593 | "AMPUTATION"[MESH] |
| #2 | 32029 | disarticulation [tiab] OR amputat* [tiab] OR amputee [tiab] |
| #3 | 38284 | #1 OR #2 |
| #4 | 206345 | "Hospitals"[Mesh] |
| #5 | 21558 | "EPIDEMIOLOGY"[MESH] |
| #6 | 544884 | epidemiolog* [ti] OR prevalence [ti] OR incidence [ti] OR frequency [ti] OR population survey [tiab] OR survey data [tiab] OR administrat* data [tiab] OR occurence [ti] OR morbidity [ti] OR mortality [ti] OR population data [tiab] OR population-based [tiab] OR community data [tiab] OR community-based [tiab] |
| #7 | 564495 | #5 OR #6 |
| #8 | 975 | #3 AND #7 |
| #9 | 59 | #8 AND (hospital [ti] OR inpatient [ti] OR hospitali* [ti] OR department [ti]) |
| #10 | 37 | #8 AND #4 |
| #11 | 81 | #9 OR #10 |
| #12 | 894 | #8 NOT #11 |
| #13 | 1 | 21388445 [PMID] |
| #14 | 135 | Related Citations for PubMed (Select 21388445) |
| #15 | 1 | 16779516 [PMID] |
| #16 | 154 | Related Citations for PubMed (Select 16779516) |
| #17 | 194 | (epidemiology [ti] OR epidemiologic [ti] OR epidemiological [ti] OR prevalence [ti] OR incidence [ti]) AND (foot [ti] OR feet [ti] OR toe [ti] OR limb [ti] OR limbs [ti] OR extremity [ti] OR extremities [ti]) AND (disarticulation [tiab] OR amputat* [tiab] OR amputee [tiab]) |
| #18 | 211 | (epidemiology [ti] OR epidemiologic [ti] OR epidemiological [ti] OR prevalence [ti] OR incidence [ti]) AND (disarticulation* [ti] OR separation* [ti] OR amputat* [ti] OR hemipelvectom* [ti] OR amputee [ti]) |
| #19 | 1296 | #12 OR #14 OR #16 OR #17 OR #18 |
| #20 | 175 | #12 OR #14 OR #16 OR #17 OR #18 Filters: Publication date from 2013/01/01 to 2015/12/31 |
| #21 | 174 | #19 AND (2013:2015[edat]) |
| #22 | 177 | #19 AND (2013:2015[crdat]) |
| #23 | 185 | #20 OR #21 OR #22 |

#### 2 MEDLINE b

Datenbase: MEDLINE (PubMed, NLM)

Search period: 2013-2014

Date of search: 19.12.2014

| **Step** | **Hits** | **Search** |
| --- | --- | --- |
| [#1](http://www.ncbi.nlm.nih.gov/pubmed/advanced) | 16593 | "Amputation"[Mesh] |
| #[2](http://www.ncbi.nlm.nih.gov/pubmed/advanced) | 78915 | disarticulation [tiab] OR separation [tiab] OR amputat* [tiab] OR hemipelvectom* [tiab] OR amputee [tiab] Filters: Humans |
| #[3](http://www.ncbi.nlm.nih.gov/pubmed/advanced) | 86216 | #1 OR #2 |
| [#4](http://www.ncbi.nlm.nih.gov/pubmed/advanced) | 317347 | "Diabetes Mellitus"[Mesh] |
| [#5](http://www.ncbi.nlm.nih.gov/pubmed/advanced) | 421556 | diabetes [tiab] OR diabetic [tiab] |
| [#6](http://www.ncbi.nlm.nih.gov/pubmed/advanced) | 480382 | #4 OR #5 |
| [#7](http://www.ncbi.nlm.nih.gov/pubmed/advanced) | 21558 | "Epidemiology"[Mesh] |
| #8 | 624051 | epidemiolog* [tiab] OR prevalence [tiab] OR incidence [tiab] OR frequency [ti] OR population survey [tiab] OR survey data [tiab] OR hospital data [tiab] OR administrat* data [tiab] OR occurence [ti] OR morbidity [ti] OR mortality [ti] OR population data [tiab] OR community data [tiab] OR hospital separation data [tiab] OR hospital amputation data [tiab] OR amputation rate* [tiab] |
| #9 | 643063 | #7 OR #8 |
| #10 | 821 | #3 AND #6 AND #9 |
| #11 | 247 | #10 AND (disarticulation* [ti] OR separation* [ti] OR amputat* [ti] OR hemipelvectom* [ti] OR amputee [ti]) |
| [#1](http://www.ncbi.nlm.nih.gov/pubmed/advanced)2 | 354 | "Amputation/epidemiology"[Mesh] |
| [#1](http://www.ncbi.nlm.nih.gov/pubmed/advanced)3 | 138 | #12 AND #6 |
| [#1](http://www.ncbi.nlm.nih.gov/pubmed/advanced)4 | 1414 | "Amputation/statistics and numerical data"[Mesh] |
| [#1](http://www.ncbi.nlm.nih.gov/pubmed/advanced)5 | 736 | #14 AND (disarticulation* [ti] OR separation* [ti] OR amputat* [ti] OR hemipelvectom* [ti] OR amputee [ti]) |
| [#1](http://www.ncbi.nlm.nih.gov/pubmed/advanced)6 | 383 | #15 AND (diabetes [tiab] OR diabetic [tiab]) |
| [#1](http://www.ncbi.nlm.nih.gov/pubmed/advanced)7 | 1 | 21388445 [PMID] |
| [#1](http://www.ncbi.nlm.nih.gov/pubmed/advanced)8 | 135 | Related Citations for PubMed (Select 21388445) |
| [#](http://www.ncbi.nlm.nih.gov/pubmed/advanced)19 | 1 | 16779516 [PMID] |
| [#2](http://www.ncbi.nlm.nih.gov/pubmed/advanced)0 | 154 | Related Citations for PubMed (Select 16779516) |
| [#2](http://www.ncbi.nlm.nih.gov/pubmed/advanced)1 | 159 | (epidemiology [ti] OR epidemiologic [ti] OR epidemiological [ti] OR prevalence [ti] OR incidence [ti]) AND (diabetes [ti] OR diabetic [ti]) AND (foot [ti] OR feet [ti] OR toe [ti] OR limb [ti] OR limbs [ti] OR extremity [ti] OR extremities [ti]) |
| [#2](http://www.ncbi.nlm.nih.gov/pubmed/advanced)2 | [72](http://www.ncbi.nlm.nih.gov/pubmed/?cmd=HistorySearch&querykey=24) | (epidemiology [ti] OR epidemiologic [ti] OR epidemiological [ti] OR prevalence [ti] OR incidence [ti]) AND (diabetes [ti] OR diabetic [ti]) AND (disarticulation* [ti] OR separation* [ti] OR amputat* [ti] OR hemipelvectom* [ti] OR amputee [ti]) |
| #23 | 793 | #11 OR #13 OR #16 OR #18 OR #20 OR #21 OR #22 |
| #24 | 77 | #11 OR #13 OR #16 OR #18 OR #20 OR #21 OR #22 Filters: Publication date from 2013/01/01 to 2015/12/31 |
| #25 | 75 | #23 AND (2013:2015 [edat]) |
| #26 | 77 | #23 AND (2013:2014 [crdat]) |
| #27 | 83 | #24 OR #25 OR #26 |

#### 3 EMBASE, Journals@OVID

Datenbase: MEDLINE (1946-2015), EMBASE (1974-2015), Journals@OVID (OVID)

Search period: 2013-2014

Date of search: 19.12.2014

| **Step** | **Hits** | **Search** |
| --- | --- | --- |
| 1 | 12599 | *AMPUTATION/ |
| 2 | 24581 | (disarticulation OR amputat* OR amputee).m_titl. |
| 3 | 28698 | 1 OR 2 |
| 4 | 124113 | *LOWER EXTREMITY/ OR *FOOT/ OR *HIP/ OR *KNEE/ OR *LEG/ OR *THIGH/ |
| 5 | 330755 | (limb* OR foot OR feet OR extrem* OR leg OR toe).m_titl. |
| 6 | 408053 | 4 OR 5 |
| 7 | 11825 | 3 AND 6 |
| 8 | 51587 | *EPIDEMIOLOGY/ |
| 9 | 1165398 | (epidemiolog* OR prevalence OR incidence OR frequency OR population survey OR survey data OR hospital data OR administrat* data OR occurence OR morbidity OR mortality OR population data OR community data OR community-based OR population-based).m_titl. |
| 10 | 1195198 | 8 OR 9 |
| 11 | 632 | 7 AND 10 |
| 12 | 316 | ((epidemiology OR epidemiologic OR epidemiological OR prevalence OR incidence) AND (foot OR feet OR toe OR limb OR limbs OR extremity OR extremities) AND (disarticulation OR amputat* OR amputee)).m_titl. |
| 13 | 487 | ((epidemiology OR epidemiologic OR epidemiological OR prevalence OR incidence) AND (disarticulation* OR amputat* OR amputee)).ti. |
| 14 | 775 | 11 OR 12 OR 13 |
| 15 | 440 | remove duplicates from 14 |
| 16 | 67 | limit 15 to yr="2013 - 2015" |
| 17 | 182 | limit 15 to em="201302-201454"  MEDLINE: 50  EMBASE: 67  Journals@Ovid: 65 |
| 18 | 80 | exportiert:  MEDLINE: 6 EMBASE: 67 Journals@Ovid: 7 |

#### 4.4 Web of Knowledge

Datenbase: Web of Knowledge (Thomson Reuters)

Search period: 2013-2014

Date of search: 19.12.2014

| **Step** | **Hits** | **Search** |
| --- | --- | --- |
| 1 | 54 | TI=(amputat* OR disarticulation OR amputee) AND TI=(epidemiolog* OR prevalence OR incidence OR morbidity OR mortality) Timespan=2013-2014  Lemmatization=On |

#### 4.5 ScienceDirect

Datenbase: ScienceDirect (Elsevier, Segmente “Medicine and Dentistry”, “Neuroscience”, “Nursing and Health professions”)

Search period: 2013-2014

Date of search: 19.12.2014

| **Step** | **Hits** | **Search** |
| --- | --- | --- |
| 1 | 0 | pub-date > 2012 and TITLE((amputat* OR disarticulation OR amputee)) and TITLE-ABSTR-KEY((epidemiolog* OR prevalence OR incidence OR frequency OR population survey OR survey data OR administrat* data OR occurrence OR morbidity OR mortality OR population data OR population-based OR community-based OR community data)) |

#### 6 CCMed, Deutsches Ärzteblatt

Datenbase: CCMed, Deutsches Ärzteblatt (DIMDI)

Search period: 2013-2014

Date of search: 19.12.2014

| **Step** | **Hits** | **Search** |
| --- | --- | --- |
| 1 | 5 | (FT=(amputat? ; disarticulation ; amputee ) AND FT=(epidemiolog? ; prevalence ; incidence ; frequency ; population survey ; survey data ; administrat? data ; (FT=(amputat? ; disarticulation ; amputee ) AND FT=(epidemiolog? ; prevalence ; incidence ; frequency ; population survey ; survey data ; administrat? data ; occurence ; morbidity ; mortality ; population data ; community data ; population-based ; community-based )) AND PY=2013 to 2015 |
