# Supplementary figures and images for: Incidence of lower extremity amputations in the diabetic compared with the non-diabetic population: A systematic review
Source: PLoS One. 2017 Aug 28;12(8):e0182081. doi: 10.1371/journal.pone.0182081 (PMC5573217; doi:10.1371/journal.pone.0182081)

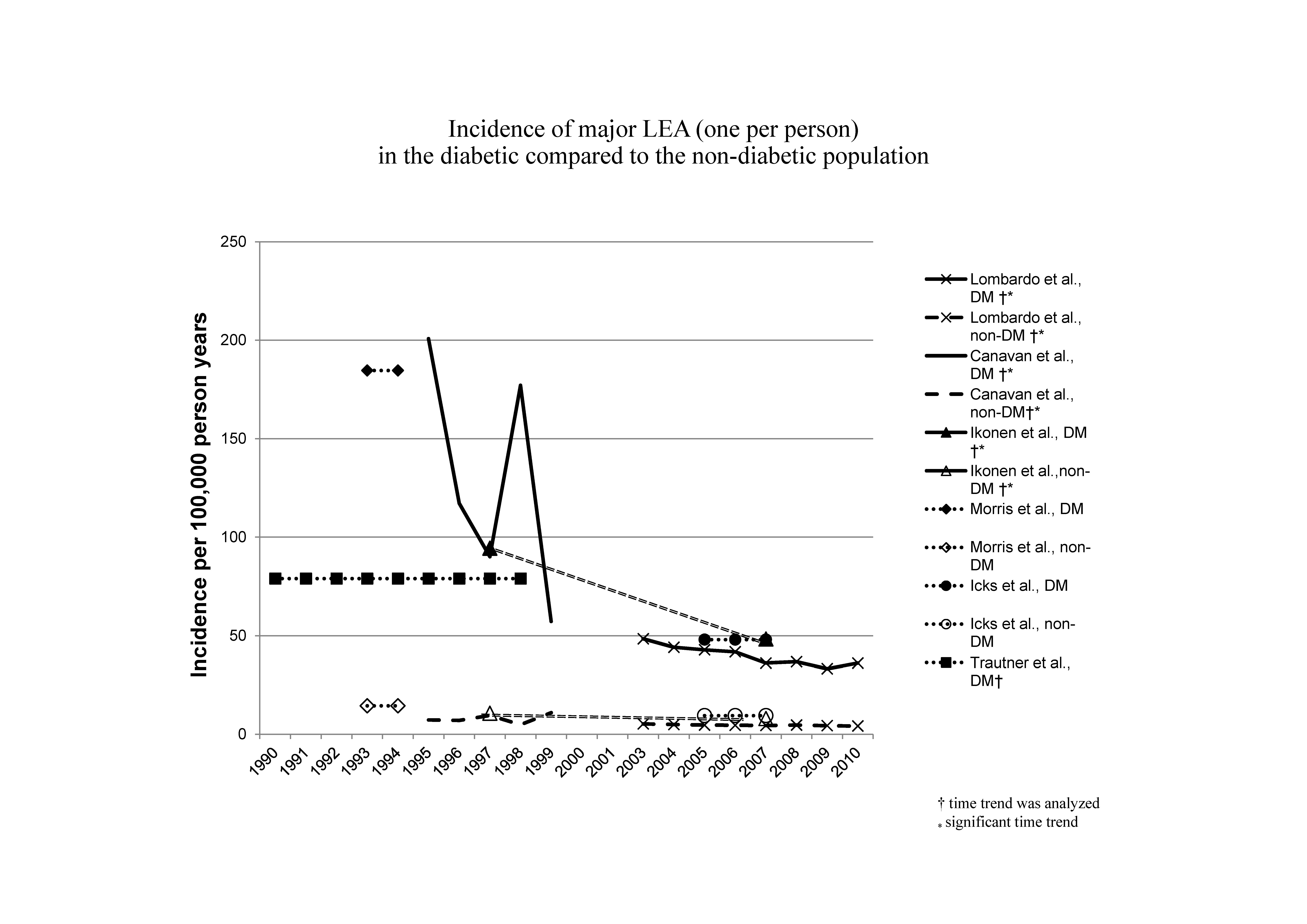

Supplement: S1 Fig — (TIFF) [file pone.0182081.s005.tiff]
